# Protein Effects on the Excitation Energies and Exciton Dynamics of the CP24 Antenna Complex

**DOI:** 10.1021/acs.jpcb.4c01637

**Published:** 2024-05-17

**Authors:** Pooja Sarngadharan, Yannick Holtkamp, Ulrich Kleinekathöfer

**Affiliations:** School of Science, Constructor University, Campus Ring 1, 28759 Bremen, Germany

## Abstract

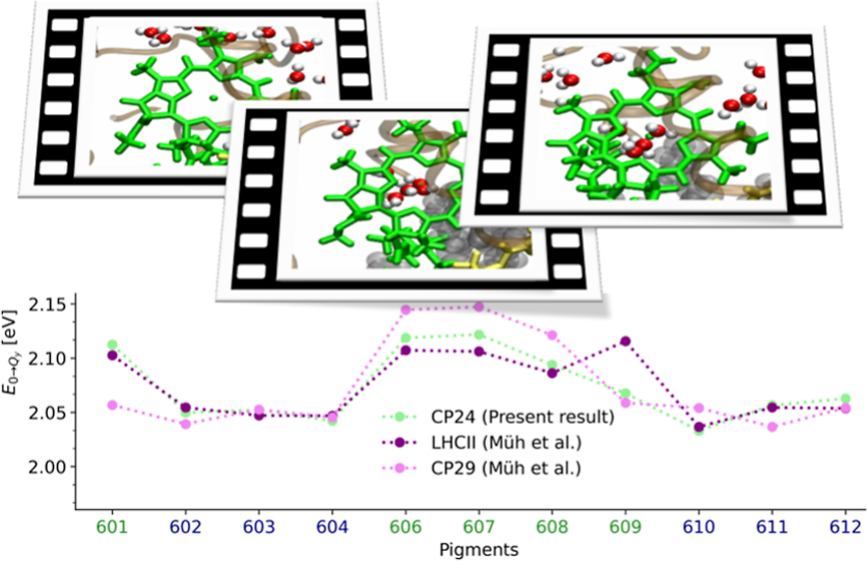

In this study, the site energy fluctuations, energy transfer dynamics, and some spectroscopic properties of the minor light-harvesting complex CP24 in a membrane environment were determined. For this purpose, a 3 μs-long classical molecular dynamics simulation was performed for the CP24 complex. Furthermore, using the density functional tight binding/molecular mechanics molecular dynamics (DFTB/MM MD) approach, we performed excited state calculations for the chlorophyll a and chlorophyll b molecules in the complex starting from five different positions of the MD trajectory. During the extended simulations, we observed variations in the site energies of the different sets as a result of the fluctuating protein environment. In particular, a water coordination to Chl-b 608 occurred only after about 1 μs in the simulations, demonstrating dynamic changes in the environment of this pigment. From the classical and the DFTB/MM MD simulations, spectral densities and the (time-dependent) Hamiltonian of the complex were determined. Based on these results, three independent strongly coupled chlorophyll clusters were revealed within the complex. In addition, absorption and fluorescence spectra were determined together with the exciton relaxation dynamics, which reasonably well agrees with experimental time scales.

## Introduction

Photosynthesis is key to sustaining life on Earth by turning sunlight into chemical energy. The photosynthetic machinery of plants and other photosynthetic organisms orchestrates this energy conversion through a series of intricate processes aided by pigment–protein complexes (PPCs).^1^ Within these PPCs, pigment molecules are anchored to a protein matrix in such a way as to form an energy funnel enabling efficient energy transfer to the reaction center where charge separation takes place.^2−5^ The pigments with high excitation energies pass the excitations to those with lower excitation energies in a cascaded fashion. The differences in excitation energy of each pigment molecule in the light-harvesting complexes (LHCs) facilitating the energy ladder arise due to different pigment types and the anisotropy in the protein environment.^6,7^ Understanding the energy map within PPCs is crucial to comprehend the excitation energy transfer (EET) and associated properties within these complexes. An enhanced understanding of these mechanisms can potentially accelerate the development of efficient artificial light-harvesting systems, bringing sustainable energy closer to reality.

**Figure 1:**
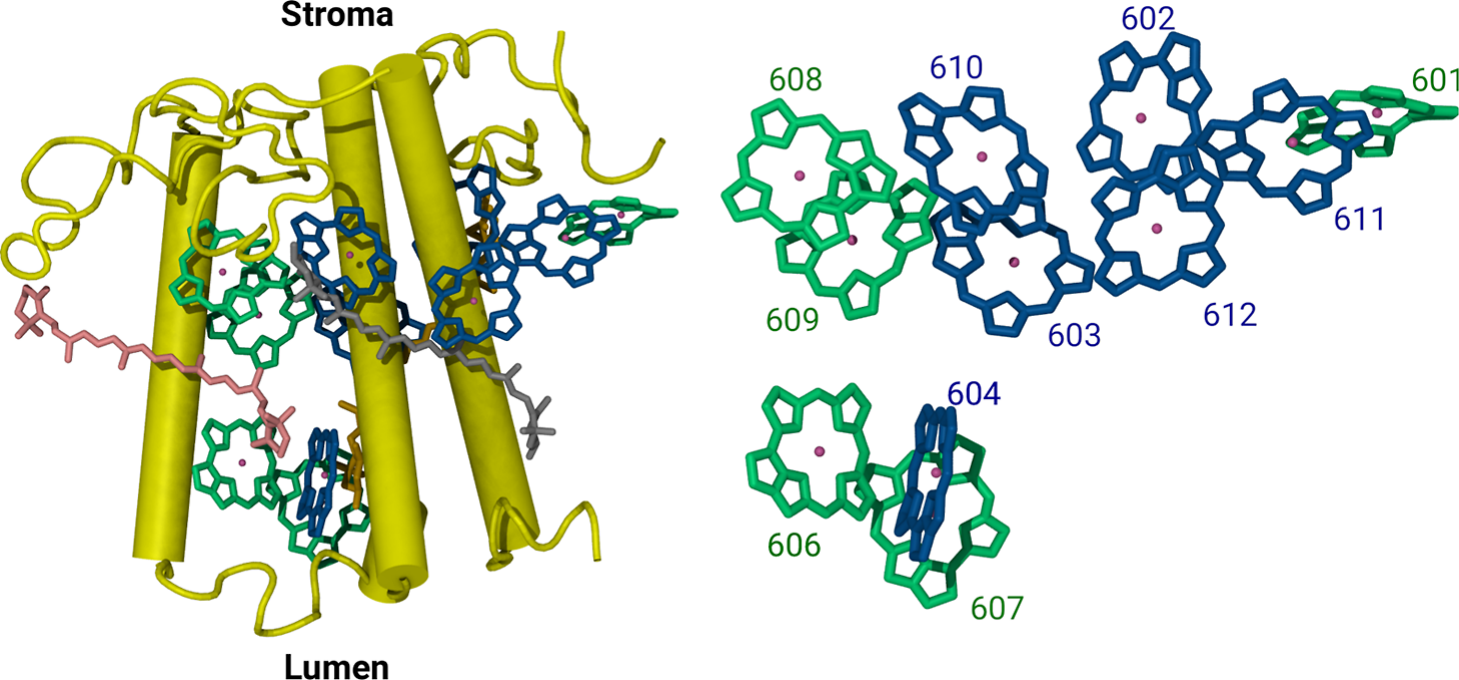
Left panel: a cartoon representation of the cryo-EM structure of the CP24 complex (PDB ID: 5XNL). The structure contains Chl-a, Chl-b and carotenoid molecules represented as sticks in different colors: Chl-a in blue, Chl-b in green, lutein in silver, violaxanthin in orange, and β-carotene in pink. Right panel: Side view of the arrangement of the Chl-a and Chl-b pigments within the complex. Only the porphyrin rings of the chlorophyll molecules without the phytyl tails are visualized, each labeled according to the nomenclature used in the Protein Data Bank (PDB).

Beyond light harvesting and EET, some of the PPCs, e.g., in higher plants, engage in photoprotection activities, shielding the complexes and the pigments therein from excessively high light intensities.^8^ In Photosystem II (PSII) of higher plants, the antenna complexes belonging to the Lhc family undergo conformational changes in response to external stimuli such as a high light intensity inducing the transition between the light harvesting and photoprotection states.^9,10^ The entirety of the antenna complexes consist of the major light-harvesting complex, i.e., the trimeric LHCII, representing the genes Lhcb1 to Lhcb3 and the minor antenna complexes CP29, CP26, and CP24 representing the genes Lhcb4, Lhbc5, and Lhbc6, respectively.^11,12^ The minor antenna complexes are located between the outer LHCII trimers and the PSII core complex. In this study, we focus on the minor antenna complex CP24 of PSII, which functions in both modes, the light-harvesting and the photoprotection mode, similar to the other complexes of the same family. A network of six chlorophyll a (Chl-a) and five chlorophyll b (Chl-b) molecules is mainly responsible for the functional properties of the CP24 complex. Sunlight initiates the excitation of the chlorophyll molecules usually to their lowest excited state, commonly referred to as the *Q*_*y*_ state, followed by a movement of the excited state energy within the system and finally toward a reaction center.^13^ In addition to the chlorophyll molecules, a β-carotenoid (BCR), a xanthophyll (XAT), and a lutein (LUT) are present in the complex as shown in Figure 1. The absence of CP24 has been shown to affect the structural integrity of PSII in the membrane.^14^ In a CP24 knockout mutant, there was a reduction in the number of LHCII trimers bound to PSII, resulting in an altered composition of the PSII-LHCII supercomplex.^15^ Plants lacking the CP24 complex were affected in their photosynthetic efficiency due to this alteration in the structural organization of PSII.^15−17^ Furthermore, the presence of CP24 in land plants and its absence in certain other species such as green algae raises questions about the importance and detailed function of this specific complex.^15,17−19^

The spectroscopic properties and photoprotective activities of CP24 complexes have been investigated in several experimental studies.^14,20−23^ However, the isolation of CP24 and similar minor complexes has been a challenge to carry out extensive experimental studies of these complexes compared to the more stable major LHCII. Purification of the complex with high concentrations of detergents resulted in loss of pigments and there was insufficient information on the pigment composition of the complex CP24.^20,24^ Early studies suggested the presence of ten chlorophyll and 2 carotenoid molecules within the CP24 complex.^14,20,21^ The recent cryo-electron microscopic (EM) structure of a PSII-LHCII supercomplex with near-atomic resolution by Su and co-workers revealed an electron density corresponding to 11 chlorophyll and 3 carotenoid molecules in the complex.^17^ Lately, 2D electronic spectroscopy measurements and calculations were performed on the LHCII-CP29-CP24 supercomplex.^23^ The calculations were based on the cryo-EM structure containing 11 chlorophyll molecules for CP24, though the site energies of the CP24 complex were adopted from the respective chlorophyll molecules in the LHCII complex. This clearly shows that a comprehensive energetic description of the CP24 complex is still lacking and further investigations are warranted. While the new structural models and spectroscopic experiments help to understand the link between structure and function, further experimental and theoretical investigations are needed to elucidate the EET processes in detail. To this end, the cryo-EM structure is a good starting point for understanding the interpigment EET within an atomistic description using the available computational tools. Extensive research, particularly on CP29 and LHCII within the Lhc family, has dissected the mechanisms of the underlying EET processes in these systems using both experimental as well as computational approaches.^25−36^ The same is, however, not yet true for the CP24 complex.

Despite significant advances in computational techniques, interpreting the excitation funnel remains a challenge due to the complexity of the system. Therefore, it is necessary to use multiscale methods that can be validated against experimental data. Efforts have been made to investigate the light-harvesting processes in LHCs through structure-based calculations, wherein theoretical models have been employed to simulate EET based on crystal structures. Such calculations typically entail modeling a Hamiltonian by incorporating structural and experimental spectroscopic information to determine, e.g., site energies and couplings.^27,30,37−42^ Despite the success of such methods in modeling Hamiltonians for many LHCs, they are not immune to inherent limitations including the lack of information on the explicit dynamics of the pigments and the surrounding proteins.^39,40^ In computing LHC excitation energies, it is crucial to consider environmental effects as they have a significant impact on the excitonic and spectroscopic properties.^43,44^ Characterizing these surroundings accurately remains a challenging task. Molecular dynamics simulations provide an atomistic representation of the system,^45^ while the energetic description is obtained through quantum mechanical methods. Recently, computational techniques have been developed to provide enhanced models for explaining complex mechanisms using quantum mechanical/molecular mechanical (QM/MM) setups.^43,44,46,47^ In this study, we utilize a combined approach of quantum mechanics/molecular mechanics molecular dynamics (QM/MM MD) and the time-dependent long-range corrected density functional tight binding (TD-LC-DFTB) level of theory^48^ to investigate the excitonic characteristics of the CP24 complex. The computations produce *Q*_*y*_ excitation energies, also known as site energies, for the pigments along a trajectory. The respective energy fluctuations result from changes in the pigment conformations but to a large degree also from that of the (protein) environment. Spectral densities, which are crucial inputs for density matrix calculations, are modeled based on the fluctuations in the site energies. The method used in this study to determine the excitation energies, TD-LC-DFTB/MM,^44,49^ has been shown to be both accurate and computationally efficient when compared to previous approaches such as Zerner’s Intermediate Neglect of Differential Orbital method with spectroscopic parameters (ZINDO/S-CIS)^50−54^ and time-dependent density functional theory^52,55^ calculations along classical MD trajectories. At the same time, the excitonic couplings are determined based on the pigment distances, mutual orientations and conformations.

The simulation trajectories provide essential information such as site energies, spectral densities, and pigment coupling, which are used as inputs for density matrix calculations or ensemble-average wave packet dynamics.^56^ The current work presents the complete Hamiltonian and exciton transfer dynamics of the CP24 complex using ensemble-averaged wave packet dynamics within the Ehrenfest formalism without back reaction. This formalism is equivalent to the so-called Numerical Integration of the Schrödinger equation (NISE).^57,58^ Furthermore, the absorption and fluorescence spectra of the CP24 complex are calculated using a Redfield-like approach^29,39,40,59,60^ and the full cumulant expansion (FCE).^61−63^ Furthermore, the resulting spectra are compared to experimental data.

## Methods

The cryo-EM structure of *Pisum sativum* at 2.7 Å served as the basis of this study. The CP24 structure, depicted in Figure 1 and employed here, was extracted from the PSII-LHCII supercomplex of *C*_2_*S*_2_*M*_2_-type (PDB ID: 5XNL).^17^ When modeling the initial structure for the MD simulations, all Chl-a and Chl-b molecules, as well as the carotenoids, were retained, while a single (1,2-dipalmitoyl-phosphatidyl-glycerole) LHG molecule was omitted. In the cryo-EM structure by Su and colleagues,^17^ no water molecules were resolved in association with the CP24 complex, i.e., no corresponding electron densities were present. Thus, no water molecules were resolved in the cryo-EM structure, nor did we model water molecules coordinating to any chlorophyll molecule in the present MD simulations. Moreover, the chlorophyll molecules in the cryo-EM structure were missing the phytyl tails. To achieve a comprehensive all-atom model of the system, the remaining parts of the chlorophyll molecules were modeled manually. To this end, a combination of GAUSSIAN,^64^ CHIMERA,^65^ and VMD^66^ was employed. In addition, this reconstruction of the phytyl tails relied on the complete chlorophyll structure available from the previously reported CP29 (PDB ID: 3PL9) and LHCII (PDB ID: 1RWT) complexes, leveraging the alignment of most chlorophyll positions when overlaying both structures with that of CP24. Additionally, missing residues were reconstructed using MODELLER.^67^

The modeled system was integrated into a POPC (1-palmitoyl-2-oleoyl-*sn*-glycero-3-phosphocholine) lipid membrane, constructed using the CHARMM-GUI web server.^68^ For the protein, the all-atom force field AMBER03^69^ was employed, and the Lipid-17 force field for the lipid bilayer. The force field parameters for Chl-a/b^69,70^ and carotenoids^71^ have been borrowed from earlier studies. Following this step, the protein–membrane system was solvated with TIP3P water molecules within a box measuring 106 × 106 × 87 Å. Moreover, NaCl ions were added to neutralize the system and to reach a concentration of 0.15 M NaCl. In total, the simulation box encompassed 103,730 atoms. The setup underwent an initial phase of energy minimization, succeeded by a 2 ns NVT equilibration at 300 K to heat up the system. During this NVT equilibration, position restraints were imposed on all components except for the water molecules using a time step of 1 fs. Following the NVT equilibration, the system underwent an 11-step NPT equilibration process, gradually releasing position restraints in several stages. Initially, for 20 ns using a 1 fs time step, position restraints were maintained on all components except the water molecules. In a subsequent 10 ns-long NPT run, again using a 1 fs time step was employed, and position restraints were released from the lipids except on the phosphorus atoms of the lipid tail. This step was followed by a 5 ns-long NPT simulation with the same time step during which position restraints were released from the phosphorus atoms of the lipid molecules. Three consecutive 5 ns NPT runs were performed by releasing the constraints one by one on the carotenoid molecules XAT, BCR and LUT. Over the next 5 ns, position restraints were limited to the protein and the chlorophyll molecules. Furthermore, in the following 5 ns, position restraints were restricted to the protein and the Mg atoms of the Chl-a and Chl-b pigments. Up to this stage, a 1 fs time step was used. The subsequent stages involved 2 ns NPT runs each, with a 2 fs time step, gradually removing position restraints on the protein, then the side chains of the protein, followed by the C-alpha atoms of the protein. Finally, a 25 ns-long run with a 2 fs time step without any position restraints was performed.

**Figure 2:**
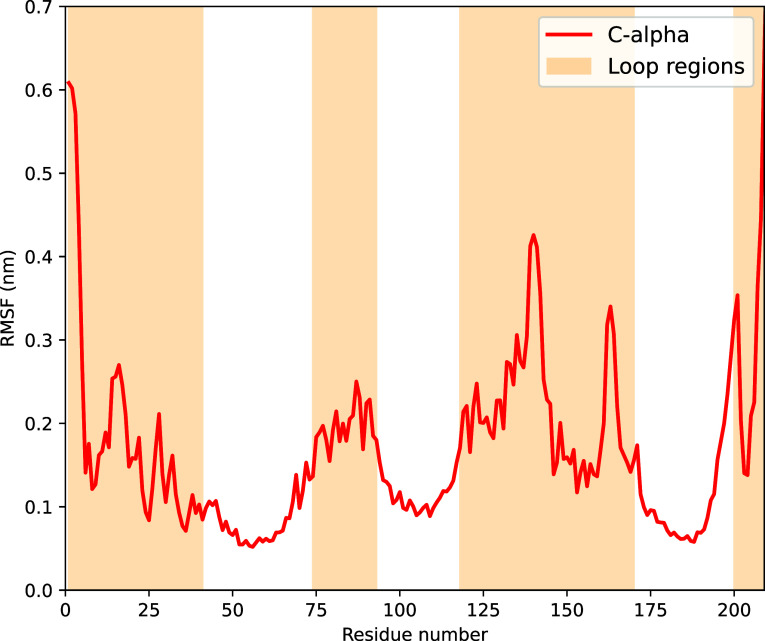
Root-mean-square fluctuations (RMSFs) of the C-alpha atoms belonging to the CP24 complex during the 3 μs-long classical MD trajectory. The loop regions are highlighted by an orange background color.

The equilibrated system was then simulated in a long unbiased MD simulation of 3 μs using the GROMACS 2021 GPU version.^72^ Throughout this simulation, noticeable fluctuations were observed in the N and C-terminal regions, along with the loop regions connecting the helices. This fluctuation pattern is highlighted in the root-mean-square fluctuation (RMSF) displayed in Figure 2, derived from 30,000 equally spaced frames encompassing the entire 3 μs-long simulation. The RMSD analysis indicates that the loop regions significantly contribute to the overall fluctuations within the complex; this data is displayed in Figure S1 in the Supporting Information. When excluding the loop regions, the structural fluctuation ranges between 0.08 and 0.25 nm. However, inclusion of the loop regions naturally amplifies the fluctuations of the C-alpha atoms of the entire complex throughout the simulations, leading to values in the range of 0.15 to 0.48 nm. The same 30,000 frames also serve as basis for the calculations of the excitonic couplings described later in this study.

To gain insight into the excited state distributions, five starting structures were selected from the 3 μs-long trajectory. This selection was made using a principal component analysis (PCA) based on the positions of the Mg atoms in the individual chlorophyll molecules, followed by a K-means clustering. In the RMSD data shown in Figure S1, it becomes clear that the C-alpha atoms exhibit significant fluctuations within the loop regions while remaining stable within the helices. These variations in the protein matrix are reflected in the relative positions of the ligands, in this case, the chlorophyll molecules. Through the interaction between the central Mg atom and the side chain of the amino acids or H-bonded water molecules, the chlorophyll molecules are anchored to the protein matrix.^73−75^ Thus, a PCA based on the relative positions of the Mg atoms in the chlorophyll molecules serves as a practical first step to identify key structures for further QM/MM MD simulations. The five frames, which are referred to as set 1, set 2, set 3, set 4, and set 5 have been extracted at time steps 182.5, 976.5, 1754.5, 2353.5, and 2887 ns as presented in Figure S2. The variations observed in the positions of Mg atoms across the five starting structures compared to the initial cryo-EM structure are illustrated in Figure S2B,C. Specifically, the Mg atoms of Chl-a 601, Chl-a 606, Chl-a 607, and Chl-b 611 have positions shifted relative to the cryo-EM structure. These chlorophyll molecules are located near the loop regions of the proteins, which undergo significant fluctuations during the simulations. These fluctuations resulted in shifts in the Mg positions, which in turn led to shifts in the positions of the chlorophyll molecules. The QM/MM MD simulations were started using these five frames as their initial coordinates. Since the site energies of the Chl-a and Chl-b molecules are a main focus of this study, the QM regions consist of the porphyrin rings of each of these chlorophyll molecules. For the QM/MM MD setup, the Chl-a consists of 82 atoms, while the Chl-b consists of 81 atoms up to the so-called C1 atom in the QM region as, e.g., used in our previous study of the complex CP29^35^ and as illustrated for a Chl-a molecule in Figure S3. The QM/MM MD simulations were performed using GROMACS 2020 combined with DFTB+ version 18.2.^76,77^ As the combined GROMACS-DFTB+ package can currently only handle one QM region at a time, we prepared 11 distinct QM/MM MD setups for the 11 chlorophyll molecules within the CP24 complex, implemented successfully across all five sets. Our QM/MM MD framework utilized the DFTB3 approach employing the 3OB-f parameter set^78^ for the QM region, alongside the classical AMBER03 force field for the MM region. The use of the frequency-corrected 3OB-f parameter set is crucial, as it more accurately describes vibrational frequencies associated with C=C, C=N, and C=O bond stretching modes, thereby enhancing the description of vibrational dynamics compared to the 3OB parameter set. To achieve equilibration in this setup, an initial 20 ps-long NPT QM/MM simulation was conducted at 300 K for all sets, employing a 0.5 fs time step. Subsequently, we generated five 1 ns-long DFTB/MM MD trajectories, with coordinates recorded every 100 fs. Additionally, two 40 ps-long simulations starting from set 1 and set 3 with a 1 fs time step were performed to generate the spectral densities of the pigment molecules. For all the resulting frames from the trajectories, excited-state calculations were carried out utilizing the TD-LC-DFTB method with the OB2 parameter set.^48^ The TD-LC-DFTB approach has been benchmarked in previous studies, showcasing its good performance in accuracy coupled with a high computational efficiency compared to DFT-based methods.^34,49^ For each pigment conformation, calculations for the 10 lowest-lying excited states were conducted using the DFTB+ package, followed by a careful extraction of the *Q*_*y*_ states along the trajectories.

## Site Energies from Different Points in the Trajectory

The aim of this study is to create a reduced model based on the fully atomistic description of the CP24 system. To this end, we introduce an excitonic tight-binding Hamiltonian of the system consisting of the site energies *E*_*i*_ of the pigments *i* and the interpigment coupling *V*_*ij*_
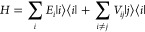
1

**Figure 3:**
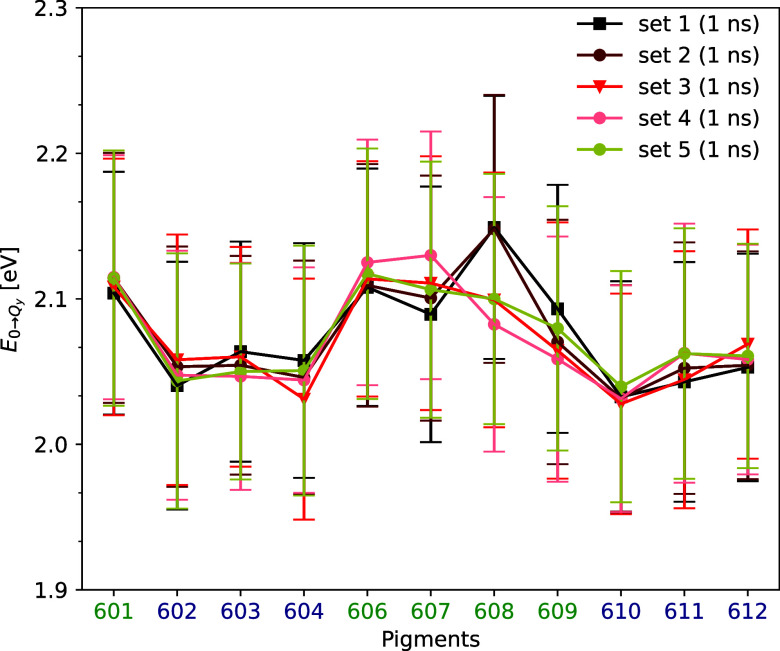
Average site energies of the 11 chlorophyll pigments within CP24 derived based on five distinct sets of 1 ns-long QM/MM MD trajectories (see text). The standard deviations are represented by the error bars, which illustrate the fluctuations in the site energies.

In this investigation, a series of five sets of 1 ns-long QM/MM MD simulations were conducted for each chlorophyll molecule to compute the site energies. These simulations started from different initial configurations extracted from the 3 μs-long MD trajectory, as described in the Methods section. The QM/MM MD trajectories resulting from these simulations were then used to compute vertical excitation energies employing the TD-LC-DFTB approach. The site energies and the corresponding fluctuations of all 11 chlorophyll molecules from these five sets, as determined by the TD-LC-DFTB calculations, are depicted in Figure 3. The respective site energy distributions resembling a Gaussian shape are shown in Figure S4. The differences in the site energies of the individual chlorophyll molecules can, besides the obvious differences between Chl-a and Chl-b, primarily be attributed to variations in the individual environments encompassing them. Significant changes in the site energies of certain pigments were observed across the different sets from the different starting points along the MD trajectory. These differences were particularly pronounced for the pigments Chl-b 607, 608, and 609. For example, looking at Chl-b 608, the differences in site energies between the sets 1 and 2 and the sets 3 to 5 is almost 0.05 eV. Considering the average differences between the site energies of the individual pigments, this difference is significant, highlighting the need to further analyze this discrepancy. On the other hand, the pigments 601, 602, 603, 610, 611, and 612 exhibit only minor differences in the average site energies across the five sets. Since the individual sets were obtained from relatively short pieces of the 3 μs-long trajectory, the environments surrounding the individual pigment molecules can change considerably between these pieces of the long MD trajectory. As a result, these changes induce shifts in the positions of the point charges within the MM region, leading to variations in the site energies between these sets. Despite the variations but also similarities in the site energies among the five sets, the Chl-b pigments have higher energy gaps from the ground to the *Q*_*y*_ state than the Chl-a molecules. This blue shift is due to the aldehyde group replacing the methyl group in the Chl-b molecules.^79^ According to the initial observations from Figure 3, the pigments with the lowest site energies are Chl-a 610 and Chl-a 604. However, the ambiguities in site energies of individual pigments among different sets make it challenging to definitively identify the excitation energy flow within the pigment network. Therefore, before a more detailed analysis of the energy transfer can be carried out, the question of the differences in the site energies of the individual pigments along the long trajectory needs to be better understood. Are these shifts due to environmental factors or due to internal changes in the chlorophyll structures themselves?

**Figure 4:**
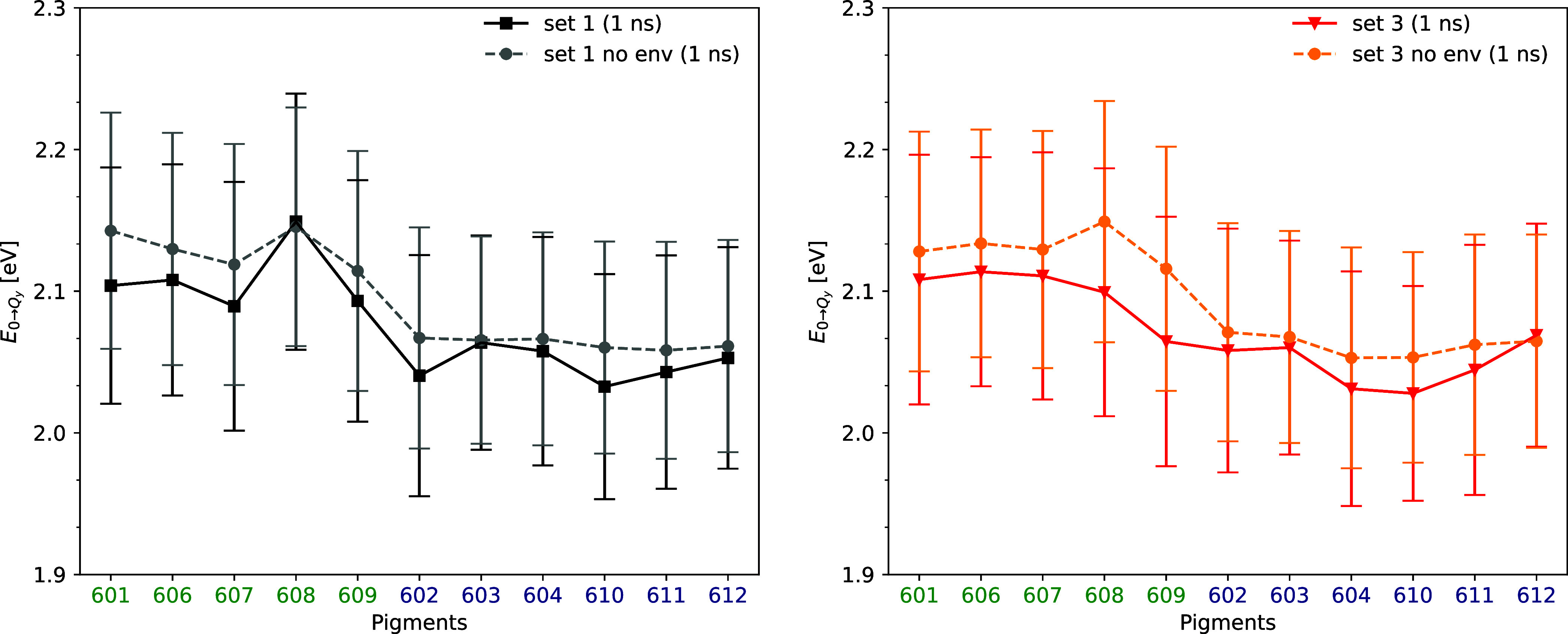
Comparison of average site energies of all chlorophyll molecules within the CP24 complex obtained from the 1 ns-long QM/MM MD trajectories belonging to set 1 (left) and set 3 (right). The error bars indicate the standard deviations of the energy fluctuations. The excitation energy calculations along the pieces of the trajectory have been executed including and excluding (“no env”) the point charges in the MM environments.

To understand the effect of the environment on the site energies, we used a trick that can only be done in simulations. Based on the same pieces of the trajectory, we performed the excitation energy calculations once including the MM point charges representing the environments and once completely ignoring these point charge surroundings. The site energies of the two sets in both scenarios are shown in Figure 4 with the Chl-a and Chl-b molecules are grouped together for better comparison. It is evident that disabling the electrostatic QM/MM coupling for these sets results in modified site energy values for all individual pigments. On the contrary, neglecting the point charges resulted in significant shifts in the site energies of certain pigments. For example, in set 1 the average site energy of Chl-b 608 including the environment is 2.149 eV and without the environment 2.145 eV while with the fluctuations are in the range of ±0.090 and ±0.084 eV, respectively. At the same time, in set 3 the same chlorophyll has a site energy of 2.099 ± 0.088 eV with environment and 2.149 ± 0.085 eV without environment. This clear disparity leads to a substantial shift of approximately 0.050 eV between set 1 and set 3, attributed to the environmental contribution. This observation underlines the limited impact of the environment on the site energies of 608 in set 1, while in set 3 a notable shift is visible. Furthermore, a comparably significant shift in site energies can be observed for Chl-b 609 between the two sets when the environment is absent. The Chl-b molecules show larger deviations in site energies compared to the Chl-a ones when the environmental charges are not included in the calculations. These changes in site energies emphasize the significance of the environment in modulating individual site energies. The chlorophyll molecules that deviate from the average site energies are also near the loop regions that contain charged amino acids, such as aspartic acid (ASP) and glutamic acid (GLU). In contrast, the pigments 603, 606, 610, 611, and 612 display quite similar site energy shifts when the environmental charges are neglected, both in sets 1 and 3. It is important to note that the environment consistently reduces the site energy in all cases. Additionally, it is essential to acknowledge that these results are based on average pigment conformations along a trajectory, rather than individual conformations. This trajectory was obtained by including all environmental atoms, regardless of how the subsequent excited state calculations are performed. Therefore, the overall site energy fluctuations here represent the combined effect of both the internal and environmental factors.

## Coordination of the Chlorophyll Molecules

To further investigate these variations based on the environment, we analyzed the vicinities of the pigments across the five sets, with a primary focus on the impact of neighboring molecules on the respective chlorophyll molecule. Within the protein-pigment complex system, specific residues within the protein matrix are responsible for anchoring the pigment molecules in their desired positions, thus preserving their functional properties in a natural context. For example, in several bilin complexes, the bilin molecules form covalent bonds with the protein matrix. In the case of chlorophyll molecules, however, their connection with the protein matrix is noncovalent. The central magnesium (Mg) atom binds noncovalently to amino acid residues in the protein or uses water molecules H-bonded to the protein backbone.^73−75^ While chlorophyll molecules typically exist in a five-coordinated state within LHCs, in the case of LH1, a six-coordinated state has been observed.^80^ Examination of the initial structures from the five sets revealed variations in the Mg coordination, particularly in cases where water coordinates, as outlined in Table 1. In this study, the coordination of the Mg atom is determined based on the Mg-ligand distances, where ligands within 2.5 Å of the Mg atom are considered to be part of the Mg coordination.^74^ For example, in the case of water molecules, the Mg-ligand distance is measured as the distance between the Mg atom and the oxygen atom of the water molecule.

**Table 1:** Coordination of the Mg Atoms of all 11 Chlorophyll Molecules in the CP24 Complex From the Initial Structures for the Five QM/MM MD Trajectories at 182, 976, 1754, 2353, and 2887 ns of the MD Trajectory^a^

	set 1	set 2	set 3	set 4	set 5
b601	Wc/Wt	Wc	Wc	Wc	Wc
a602	GLU	GLU	GLU	GLU	GLU
a603	HIS	HIS	HIS	HIS	HIS
a604	Wt	Wt	Wt	Wt	Wt
b606	GLN	GLN	GLN	GLN	GLN
b607	Wc/Wt	Wc/Wt	Wt	Wt	Wt
b608			Wc/Wt	Wc/Wt	Wc/Wt
b609	GLU	GLU/Wc	GLU/Wc	GLU/Wc	GLU/Wc′
a610	GLU	GLU	GLU	GLU	GLU
a611	Wc/Wt	Wc/Wt′	Wc/Wt″	Wc/Wt″	Wc/Wt″
a612	HIS	HIS	HIS	HIS	HIS

a The terms “Wc” and “Wt” refer to water molecules coordinating from the same side of the phytyl tail (similar to a cis coordination) and to water molecules coordinating from the side opposite to the phytyl tail (similar to a trans coordination), respectively. Additional primes in the notation of the water molecules indicate that the respective water molecule has been swapped.

**Figure 5:**
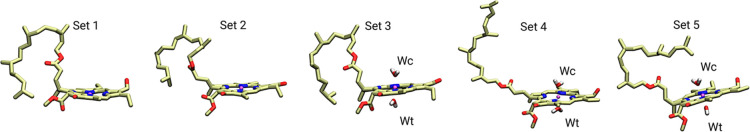
Dynamic coordination of water molecules within 2.5 Å nm of Mg in Chl-b 608 over the 3 μs-long MD simulation.

**Figure 6:**
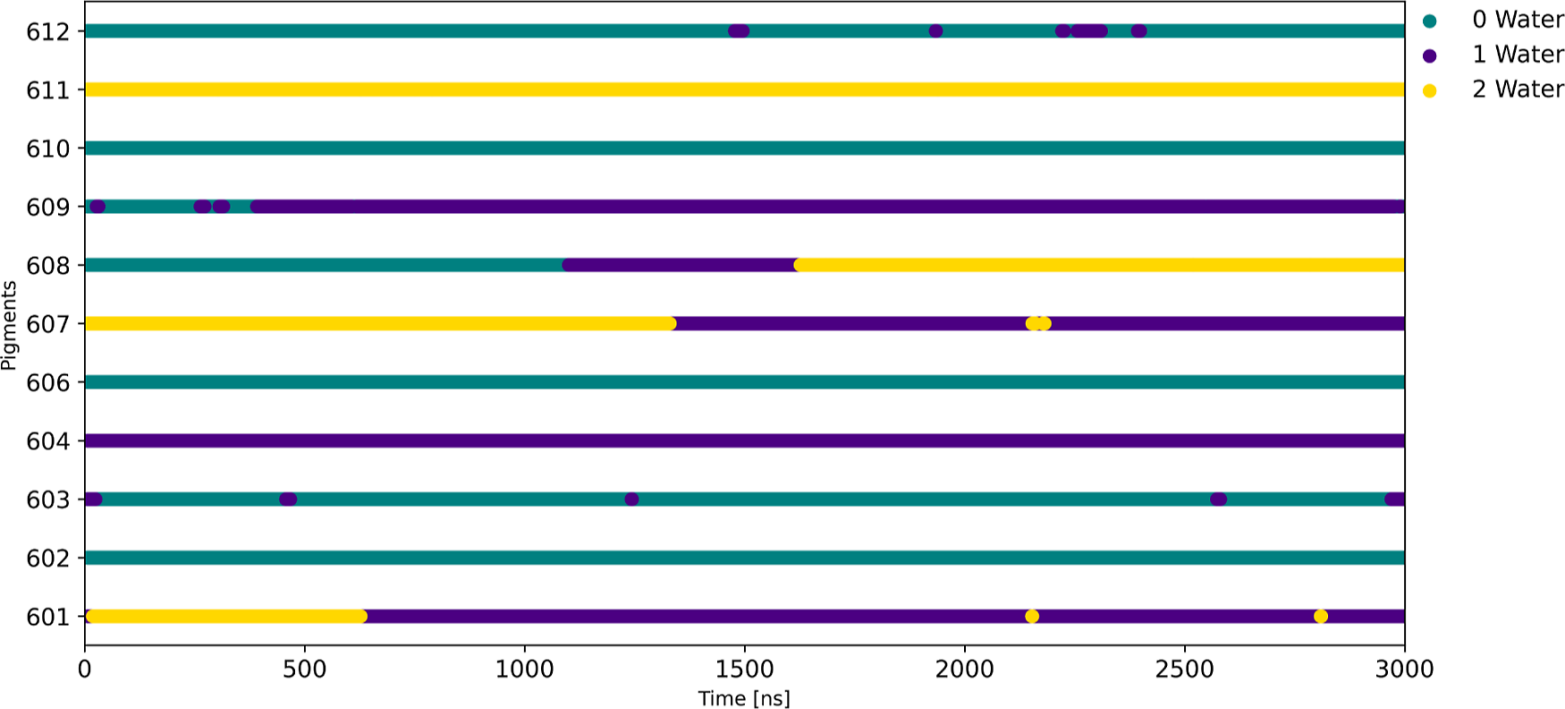
Dynamic coordination of water molecules within 2.5 Å of Mg in chlorophyll molecule 608 from 5 initial configurations. The water molecules are coordinated to the Mg atom in sets 3, 4 and 5.

During the 3 μs-long MD simulation, we observed some dynamics in the coordination state for the central Mg atom in some chlorophyll molecules. In the cases where water molecules predominantly act as coordinating ligands, the coordination state of the Mg atom fluctuates between five and six. The time evolution of the coordination numbers for each of these pigments is depicted in Figure 6. As an example, during the initial stages of the simulation Chl-b 601 exists in a six-coordinated state but at a later point in time transitions to a five-coordinated state with a water ligand binding to the same side as the phytyl tail as represented in Figure 6 and also detailed in Table 1. Similarly, Chl-b 607 experienced changes in the number of coordinating water molecules over the course of the simulation. In the case of Chl-a 611, two water molecules are bound to the Mg atom along the full trajectory. It is worth noting that during the initial NVT equilibration phase, water molecules began coordinating the pigments a604, b607, and a611. Subsequently, during the first NPT equilibration, a water molecule coordinated with pigment b601. Thus, the coordination of these pigments occurred during the early stages of equilibration. Surprisingly, pigment Chl-b 608 does not have any coordination in sets 1 and 2 as can be seen in Figure 5. Also, in the cryo-EM structure,^17^ no coordinating water molecules have been resolved near the Mg atom of Chl-b 608. In the MD simulation, after almost 1 μs one and after about 1.5 μs two water molecules coordinate to that chlorophyll pigment. When looking at crystal structures of the very similar complexes LHCII (PDB ID: 1RWT, 2BHW) and CP29 (PDB ID: 3PL9), the corresponding chlorophyll molecule does have a water molecule coordinated to the respective Mg atom.^11^ This fact strongly suggests that indeed a water molecule should coordinate to Chl-b 608 in order to exist in a five-coordinated state in the CP24 complex. All other chlorophyll molecules had either a six- or a five-coordination from the start of the simulation, but for this particular chlorophyll, the water coordination occurred only later in the MD simulation. Moreover, Chl-b 609, originally coordinated to a glutamic acid, changed to a six-coordinated state in sets 2 to 5. Although the similar complexes do not suggest a six-coordinated state, it is something that came up as a result of the 3 μs-long MD simulations. In Figure 4 one can see that the pigments with a large shift in site energies are 608 and 609, both of which have a water molecule coordinated in the sets with lower site energies. These findings, which will be further detailed below, are not the first of their kind but are consistent with earlier studies. In one of these previous investigations, it has been shown that the Mg-water coordination causes a redshift in absorption spectra with a larger effect in the B-band.^36,81^ Although the main difference between sets 1 and 3 in the configuration of the respective environments around Chl-b 608 is the appearance of water molecules forming a six-coordinated state, the electrostatic effect of this water coordination does not affect the site energy. According to the site energy values in both sets with and without the environment as shown in Figure 4, the shift in site energy is due to the electrostatic effect of the environment. Of all the different components in the environment, the effect of the protein matrix is prominent in both cases.

**Figure 7:**
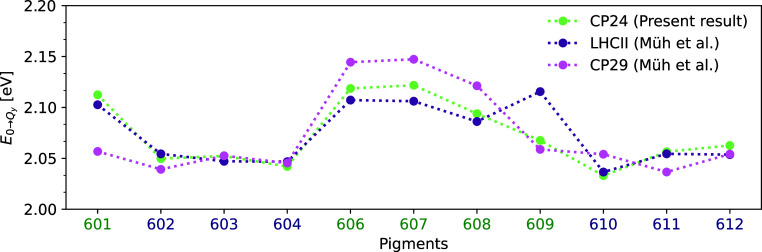
Average site energies of the pigments in the CP24 complex along the 1 ns-long trajectory using TD-LC-DFTB. The present findings (average of sets 3, 4 and 5) are compared to the site energies of the CP29 and LHCII complexes as determined by Müh et al.^27^ based on the crystal structure. The LHCII site energies are shifted by 0.203 eV and the CP29 energies by 0.207 eV for better comparison.

In a next step, we compared the present average TD-LC-DFTB site energies (average of sets 3, 4 and 5) of the CP24 complex with those based on the crystal structure of the CP29 and LHCII complexes obtained using the Poisson–Boltzmann/quantum chemical (PB/QC) approach by Müh et al.^27^ The data is shown in Figure 7 including shifts for the absolute energies of the CP29 and LHCII complexes for a better comparison. The unshifted data is shown in Figure S5. This comparison has been chosen since the site energies of the CP24 complex have not been determined earlier, and the CP29 and LHCII complexes are very similar in structure.^17,20^ Nonetheless, minor structural differences can have rather drastic effects on the spectroscopic properties. Neglecting the fact that the present absolute excition energies are likely overestimated due to shortcomings of DFT-based approaches, quite some similarities are visible in the relative site energies. It should be noted that one of the pigments in the CP29 complex has a different numbering, while the numbering of the pigments in CP24 and LHCII is the same. The pigment at the same position as Chl-a 601 in CP24 and LHCII is named Chl-a 615 in the CP29 system.^35^ Therefore, the site energy of Chl-a 615 from Müh et al. is used as the site energy of Chl-a 601 in Figure 7, S5 and S6. The average site energies of CP29 calculated using the TD-LC-DFTB method in the previous study^35^ are compared to the CP24 site energies and to those from the PB/QC method as depicted in Figure S6. As can be seen in Figure 7 and S5, the average site energies of the pigments based on sets 3, 4, and 5 of CP24 show a similar trend to those of the LHCII complex, except for Chl-b 609. Pigment 609 is a Chl-b molecule in both LHCII and CP24 complexes, whereas it is a Chl-a molecule in the CP29 complex. In the CP24 complex, the site energy of this pigment shows a distinct shift, in contrast to the behavior observed for the LHCII pigment, although both pigments bind to a glutamic acid residue in their respective complexes.^11^ The structural difference observed in the pigment 609 compared to the dynamics of CP24 is the additional water coordination on top of the glutamic acid. While the site energies for the CP24 and LHCII complexes show a very large similarity except pigment 609, there are larger differences for several pigments between the complexes CP24 and CP29.^20^Figure S6 provides a more effective comparison of site energies between CP29 and CP24, as the same level of theory has been applied. The TD-LC-DFTB site energies for most pigments in CP29 are similar to those in the CP24 complex, with the exceptions of pigments 601, 606, and 608. As previously noted, pigment 601 is a Chl-a pigment in CP29 and a Chl-b molecule in CP24. It has been reported that the trend of the site energies between the DFTB/MM MD trajectory-based TD-LC-DFTB and the structure-based PB/QC energies in CP29 parallels the trends observed in Figure 3 of Maity et al.,^35^ particularly for Chl-b 606 and Chl-b 608 as shown in Figure S6. Despite using the same theoretical framework, there are more pronounced differences in the site energies between CP24 and CP29 than those observed between LHCII and CP29. Therefore, the CP24 complex is likely to have excitonic properties more similar to those of LHCII than to those of the CP29 complex, however, this needs to be analyzed in more detail, including the excitonic couplings. In these comparisons, we have used the average of sets 3 to 5 representing the cases with proper water coordination not yet present in sets 1 and 2. The same average is also used for further comparisons with literature values and calculations of couplings and spectroscopic properties below. Note that in previous studies, we examined MD trajectory frames separated by only a few nanoseconds and found reasonable agreement between these sets.^32,34,35,47,82^ However, when using low-resolution structures without the proper water ligands, as in this case, extra care must taken to properly equilibrate the system and to ensure that water molecules can move to locations that are not easily accessible. This is especially important when looking at electronic properties, as was done here, since these can be particularly sensitive to small structural changes and proper placement of water molecules.

## Spectral Densities

The so-called spectral density is a quantity describing the frequency-dependent coupling of the primary system to its environment. In the present case, the primary system consists of coupled two-level systems mimicking the ground and excited *Q*_*y*_ states of the individual chlorophyll molecules. The environment is given by the protein matrix, the lipids, the water, and ions but also the internal vibrational modes of the pigments since these are not part of the primary system. Spectral densities are calculated as cosine transformations of the site energy autocorrelation functions. The theoretical background for calculating the spectral densities has been detailed elsewhere.^43,44,50,83^ Given the fact that each chlorophyll molecule in the CP24 complex is situated in a distinct environment, the resulting fluctuations in the site energies are slightly different for each pigment. The spectral densities of the individual chlorophyll molecules therefore reflect these nuanced energy fluctuations, as delineated in Figure S7. Even with their structural differences, the spectral densities of the Chl-a and Chl-b molecules are quite similar, as already found for the case of the CP29 complex.^35^

**Figure 8:**
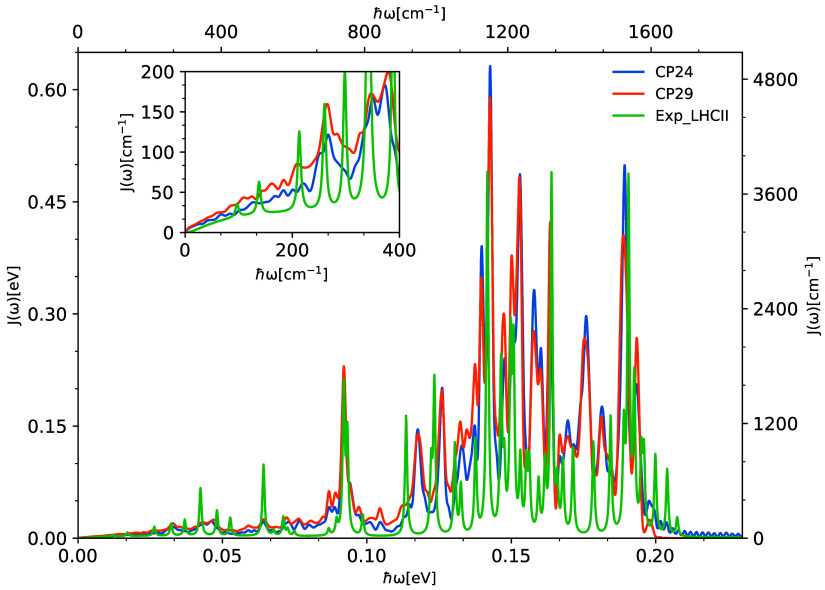
Comparison of the spectral densities of the CP24 complex averaged over all pigments in the system. The result is based on 60 ps-long QM/MM MD trajectories from sets 1 and 3. The spectral density for the CP29 complex has been obtained in a similar manner.^35^ Also shown is the experimental spectral density of the LHCII complex.^37^

Spectral densities for all chlorophyll molecules within the CP24 complex were computed based on the site energy fluctuations derived from 60 ps-long QM/MM MD trajectories of set 1 and set 3. In Figure 8, the average spectral density for the 11 chlorophyll molecules from these trajectories is compared to that of the CP29 complex^35^ and the experimental spectral density of LHCII based on the parameters from fluorescence line narrowing experiments at 77 K given in Tables 1 and 2 of ref (37). Similar to earlier studies based on the same DFTB/MM procedure for the LHCII,^34^ the CP29,^35^ and the CP43 complexes^82^ also in the case of the CP24 pigment–protein complex, the calculated average spectral density agrees well with the experimental spectral density of the LHCII complex. No experimental results are available for the other complexes. Since the complexes CP24, CP29, and LHCII are quite similar, and share important characteristics, it is not too surprising that the widths and overall line shapes of the spectral density peaks coincide reasonably well. It is noteworthy that the number of pigments and the Chl-a to Chl-b ratio differ among these complexes. Furthermore, the slight variations in the site energy fluctuations caused by the differences in the immediate surroundings of the individual chlorophyll molecule reflect on the spectral densities, hence the spectral densities of the individual pigments vary slightly. Averaged over all pigments of one complex, however, some of these differences apparently get washed out.

**Table 2:** Time-Averaged System Hamiltonian of the CP24 Complex Based on the Exciton Coupling Values Extracted Along the 3 μs-Long Classical MD Trajectory and the Average Site Energies From the 1 ns-Long QM/MM MD Simulations of Sets 3 to 5^a^

	b601	a602	a603	a604	b606	b607	b608	b609	a610	a611	a612
b601	**17037.9**	**35.6**	–0.6	–0.8	–0.7	–0.8	1.9	1.8	–3.5	3.1	5.8
a602	**35.6**	**16532.1**	**50.5**	4.2	3.8	4.4	–5.6	–21.6	–5.7	–2.2	7.3
a603	–0.6	**50.5**	**16553.1**	–5.7	–8.2	–8.0	2.1	**58.3**	6.5	–0.4	–2.4
a604	–0.8	4.2	–5.7	**16469.8**	**46.7**	21.7	–2.3	0.5	–3.0	–2.0	1.6
b606	–0.7	3.8	–8.2	**46.7**	**17088.1**	22.8	–4.1	4.2	–2.1	–1.7	1.6
b607	–0.8	4.4	–8.0	21.7	22.8	**17113.1**	–2.3	0.2	–1.0	–1.7	1.7
b608	1.9	–5.6	2.1	–2.3	–4.1	–2.3	**16888.0**	25.1	**51.4**	3.9	–1.9
b609	1.8	–21.6	**58.3**	0.5	4.2	0.2	25.1	**16676.3**	–0.5	2.5	–0.4
a610	–3.5	–5.7	6.5	–3.0	–2.1	–1.0	**51.4**	–0.5	**16397.1**	–26.3	**39.9**
a611	3.1	–2.2	–0.4	–2.0	–1.7	–1.7	3.9	2.5	–26.3	**16586.7**	**89.4**
a612	5.8	7.3	–2.4	1.6	1.6	1.7	–1.9	–0.4	**39.9**	**89.4**	**16636.4**

a The site energies and the coupling values are given in units of cm^–1^ while values larger than 30 cm^–1^ are highlighted in bold.

The inset in Figure 8 shows that the low-frequency component of the average spectral density exhibits a good but not perfect alignment with the experimental spectral density of the LHCII complex. The spectral density in this frequency domain is due to the electrostatic coupling between the excitation energies and the environment.^53^ The peaks in the low-frequency region from 200 to 400 cm^–1^ can, however, only be reproduced to a limited degree. Comparing the average computed spectral densities for the CP24 and CP29 complexes, it becomes evident that in the low-frequency region, the amplitude of CP24 is lower than that of CP29, while in the high-frequency region the CP24 peaks are higher. It has to be further analyzed in the future how statistically relevant these changes are and if they have a significant effect on the exciton dynamics, which is mainly controlled by the low-frequency components of the spectral densities. As already shown for the LHCII,^34^ the CP29,^35^ and the CP43 complexes,^82^ the high-frequency section of the computed average spectral density of CP24 shows a good agreement in peak positions, albeit they are slightly broader than the experimental peaks. These high-frequency peaks arise from intramolecular vibrations involving C=C, C=N, and C=O bond stretching, which are well reproduced using the DFTB3 together with the 3OB-f parameter set which is optimized for vibrational features.^47^ While obtaining an accurate spectral density is not our ultimate goal while studying LHCs, it is rewarding to see that for this property the agreement between theory and experiment is quite good and has considerably improved over the past few years.

For later use, we also introduce the reorganization energies λ_*m*_ of the individual pigment molecules, given by
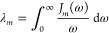
2

The respective values are listed in Table S1.

## Hamiltonian

The interactions between the pigments are of key importance not only for the exciton dynamics but also significantly influence the spectral features of LH complexes. The coupling values are indeed very sensitive to conformational changes in the protein structure. To obtain a proper sampling of the coupling values, we computed the excitonic couplings between the chlorophyll molecules in the CP24 complex along the 3 μs-long MD simulation. Utilizing parts of the (much shorter) QM/MM trajectories for coupling calculations is limited due to the current implementation of the QM/MM approach, which accommodates only one QM region, representing one pigment, at a time. To this end, the couplings *V*_*ij*_ in eq 1 have been calculated using the TrEsp (transition charges from electrostatic potential) method developed by Renger and co-workers^84,85^ based on the 3 μs-long MD simulation. Using this approach, the coupling between pigments *m* and *n* is given by
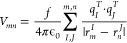
3where *q*_*I*_^*T*^ and *q*_*J*_^*T*^ denote the transition charges of atoms *I* and *J* from the respective donor *m* and acceptor *n* molecule. We have adopted the transition charges for the heavy atoms of the Chl-a and Chl-b pigments from a previous study.^35^ These transition charges were rescaled to reproduce the experimental transition dipole moments of 5.7 D for Chl-a and of 4.6 D for Chl-b.^86^ Environmental effects on the excitonic coupling values have been approximated by the distance-dependent screening function *f* by Curutchet et al.^87^
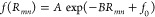
4where *R*_*mn*_ denotes the distance between the centers of the two respective molecules and the parameters *A*, *B* and *f*_0_ have the values 2.68, 0.27 and 0.54, respectively. The average site energies used for representing the time-averaged Hamiltonian were obtained by averaging the site energies from sets 3 to 5. The resulting time-averaged Hamiltonian for the CP24 complex in site basis is detailed in Table 2, which can be used for further research on the CP24 complex.

**Figure 9:**
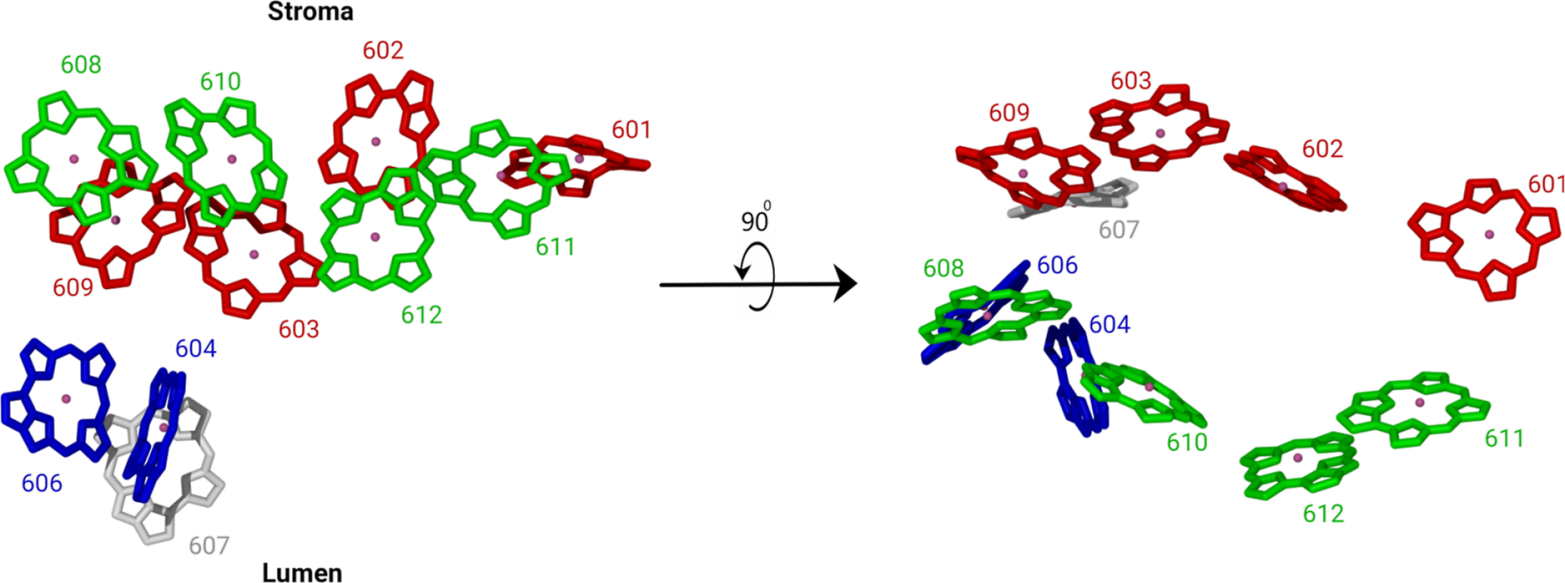
Based on the excitonic coupling values, the chlorophyll molecules in the CP24 complex are arranged in different clusters, as indicated by the different colors. Left: side view, right: top view.

While the site energies have been discussed above, here we focus on the excitonic couplings. The highest coupling value occurs between Chl-a 611 and Chl-b 612. Additionally, there is a rather strong coupling between Chl-a 612 and Chl-a 610, with the latter having the lowest site energy among all pigments and being positioned close to the LHCII-CP24 interface. Chl-a 610 in turn is strongly coupled to Chl-b 608, which makes a cluster of three Chl-as and one Chl-b. This cluster, which is especially characterized by the large contribution of Chl-a 610 to the lowest excitonic state (as shown in Figure S8), makes the 608-610-611-612 ensemble prominent in the first excitonic state. Similarly, another cluster consisting of the four coupled chlorophylls 601-602-603-609 can be identified. Moreover, Chl-a 604 and Chl-b 606 can be seen as a separate cluster. In contrast, Chl-b 607 is not strongly coupled to any of the other pigments and has coupling values of about 20 cm^–1^ to Chl-a 604 and Chl-b 606 while the three previously discussed clusters have coupling values of greater than 30 cm^–1^ among their constituents. These clusters are depicted in Figure 9. The pigments Chl-a 610 and Chl-a 604 with low site energies are located in the region separating LHCII and CP24, suggesting that these chlorophylls are involved in the energy transfer between these two complexes. Structurally, the chlorophyll molecules 604, 606, and 607 are located toward the lumenal side of the membrane while the chlorophylls 601, 602, 603, 608, 609, 610, 611, and 610 are located toward the stromal side of the membrane. These sets of chlorophyll molecules residing at the different edges of the membrane are well separated with an average distance between the chlorophyll molecules of about 20 Å (see Figure S9 for Mg–Mg distances) leading to small excitonic couplings between these pigments. Strong excitonic couplings are observed between the pigments within these clusters, as is evident from the coupling matrix. The coupling between the two clusters on the stromal side is quite large, with values greater than 20 cm^–1^ between the pigment pair Chl-b 608: Chl-b 609. Thus, one could also assume that all pigments on the stromal side of the complex form a single cluster consisting of two subclusters with even larger intrasub-cluster couplings.

Based on the coupling values and site energies, potential energy transfer pathways between the pigment molecules can be proposed. In an energy funnel, the pigment with the highest excitation energy is expected to transfer its energy to the nearest neighbor with a lower excitation energy if a non-vanishing coupling exists between the two chromophores, and so on. Due to the robust network of couplings between the pigments on the stromal side of the membrane, a potential energy transfer pathway with a large participation of these pigments can be expected. The pigments with the highest excitation energies are Chl-b 606 and Chl-a 607. Since pigment 607 belongs to the 604–607 cluster on the luminal side of the membrane, an energy transfer to the pigments located on the other side of the membrane is quite limited. In addition, Chl-b 606 is not strongly coupled to the pigments on the stromal side, so its contribution to the energy transfer likely negligible. The chlorophyll molecule with the next highest site energy is pigment 601, which offers several possibilities for energy transfer to other nearby pigments. Therefore, it seems obvious that most of the EET occurs in and between the clusters 608-610-611-612 and 601-602-603-609. A more detailed analysis of the EET based on an ensemble-averaged wave packet dynamics is given below.

## Absorption and Fluorescence

Based on the calculated time-averaged Hamiltonian, spectral densities, and transition dipole moments (extracted from the TD-LC-DFTB calculations of the 1 ns-long trajectories and given in Table S2) one can determine the absorption and fluorescence spectra of the CP24 complex. In the present study, we employ the FCE formalism to calculate the absorption spectra as given by eq 5 in the study Cupellini et al.^63^ In addition, we use a Redfield-like formalism because we want to calculate the absorption as well as the fluorescence spectrum with the same approach. However, it is known that Redfield-like theories certainly have their limitations and have been shown to be problematic for some LH complexes.^63,82,88^ The excitation energies and the respective transition dipole moments determine the energetic positions and intensities of the spectroscopic features. The broadening of these lines, i.e., the line shapes, of these contributions to the spectra are determined by the spectral densities in a way detailed below. The Hamilton operator of the LH system has been defined in eq 1 and its numerical values for the CP24 system are given in Table 2. Diagonalization of this Hamiltonian yields the excitonic states and energies. Each excitonic state |μ⟩ can be written as a linear combination of the states |*i*⟩ localized at the pigments *i*
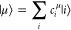
5where *c*_*i*_^μ^ denotes the contribution of *i*-th site to the excitonic state |*m*⟩. The same coefficients can be used to transfer the transition dipole moments *d*_*i*_ from the site into the excitonic picture
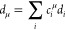
6

Together with the spectral densities, this information is already enough within the Markovian Redfield theory to determine the transfer rates between the eigenstates for the case ω_μν_ > 0
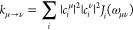
7while for ω_μν_ < 0, the principle of detailed balance can be employed together with the inverse temperature β = 1/*k*_B_*T* as
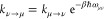
8In this equation, ω_μν_ denotes the transition frequencies between exciton states μ and ν given by (*E*_μ_ – *E*_ν_)/ℏ. Using the obtained rate expressions, the lifetime of the excitonic state τ_μ_ can be determined as
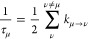
9

Moreover, the line shape function in site basis is given by
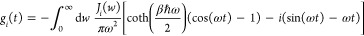
10which can be transformed into the excitonic picture using
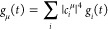
11

In turn, this expression can be used to evaluate the absorption *D*_μ_(ω) and the fluorescence line shapes 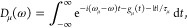
12
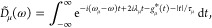
13where λ_μ_ denotes the reorganization energies in the excitonic picture^51,89^
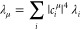
14

Having obtained all these ingredients, the absorption α(ω) and fluorescence spectra *I*(ω) within the Redfield approximation are given by^39,40,59^
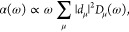
15
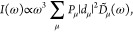
16where *P*_μ_ denotes the Boltzmann factor
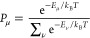
17

**Figure 10:**
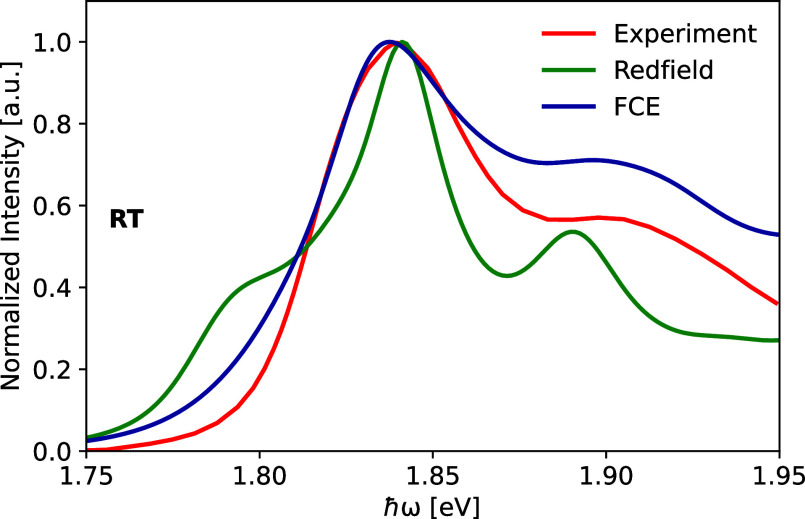
Absorption spectra of the CP24 complex based on the FCE and Redfield methods compared to the experimental spectrum at room temperature.^20^ The FCE and the Redfield data have been shifted by 0.10 and 0.16 eV, respectively, toward lower energies to match the position of the main experimental peak.

**Figure 11:**
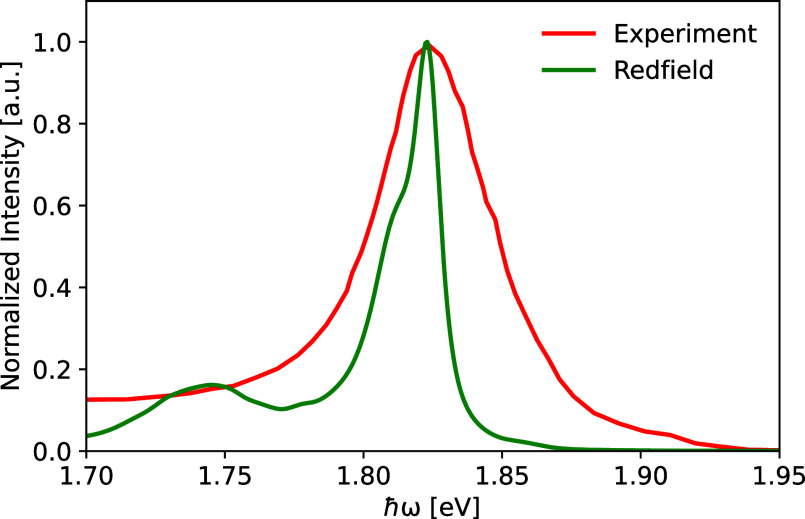
Fluorescence spectra of the CP24 complex based on the Redfield method compared to the experimental results.^20^ The Redfield data has been shifted by 0.16 eV toward lower energies to match the experimental peak position.

The calculated absorption and fluorescence spectra are compared to the experimental counterparts^14,20^ and given in Figures 10 and 11, respectively. It is essential to note, however, that the samples used in the experimental spectroscopic measurements are from a different organism, namely *Arabidopsis thaliana*.^20^ The CP24 complex of that organism in the aforementioned experiments consists of only five Chl-a and five Chl-b pigments as well as two xanthophyll molecules. In contrast, the CP24 complex of the organism *P. sativum*, from which the cryo-EM structure was used here, contains 11 chlorophyll and three carotenoid molecules.^17^ This difference in the chlorophyll count might significantly impact the overall spectral properties of the CP24 complex and therefore a partial reason for the differences in the spectra. For example, a case study involving chlorophyll knockout mutants resulted in notable differences in the absorption spectra, suggesting that even a single chlorophyll molecule can significantly affect spectra.^20^ Considering this fact, it is important to acknowledge that the spectroscopic comparison presented in this study certainly has its limitations.

The calculated spectra were shifted toward lower energies to align with the main peak positions of the experimental spectra. This adjustment allowed a better comparison of spectral features between the calculated and measured spectra, compensating for the overestimation of excitation energies in DFT-based theories. In addition, the peak heights of the experimental and calculated spectra were normalized to unity. The absorption spectrum calculated from FCE formalism is in quite good agreement in terms of the spectral width of the main peak. The vibrational sideband is too high compared to the experimental result, similar to our findings for the CP29 and CP43 complexes.^35,82^ As expected, the non-Markovian FCE formalism yields a more accurate result for absorption spectrum than the Redfield approximation. As explained above, we used the average site energies from sets 3 to 5 for the calculation of the absorption spectrum to avoid shifts due to the missing coordination of chlorophyll molecules.^36^ To enhance the accuracy of the Redfield results, one can choose to separately analyze the three distinct clusters and obtain absorption spectra, as demonstrated in Figure S10. This approach involves disregarding the weakest coupling between pigments when computing the spectra.^90^ However, despite implementing this strategy, only a marginal improvement was observed in the spectra with the disappearance of lower energy peaks evident in the Redfield data shown in Figure 10. Additionally, the absorption spectrum was computed at 77 K and is depicted in Figure S11. Furthermore, the fluorescence spectrum obtained using the Redfield approximation shows a significantly lower width compared to its experimental counterpart. This result is possibly due to the limitations of the Redfield approach in the presence of weakly coupled clusters within the system.^39,82,90^ Moreover, no static disorder was included in the present work. The QM/MM simulations employed in this study are already computationally demanding, which limits the number of such simulation which can be done with reasonable numerical effort. One could also consider extracting the static disorder from the MD simulations from uncorrelated snapshots^43,59,83,91^ but this is beyond the scope of this study. Nevertheless, the agreement of the experimental and computational absorption spectra indicates that the parameters obtained through the present molecular simulations are very reasonable.

## Exciton Dynamics

The time-dependent Hamiltonian of the system was used to generate the exciton dynamics in the system. Since we are studying an isolated CP24 complex, only limited information on the exciton dynamics under physiological conditions within the PSII supercomplex can be extracted. The data shown here can only be related to the exciton dynamics within the isolated complex. To this end, individual pigment molecules were initially excited and the time evolution of the exciton wave function was determined using an ensemble-averaged wave packet scheme called NISE (numerical integration of the Schrödinger equation). Denoting an excitonic state within the single-exciton manifold by |ψ_*s*_(*t*)⟩ represents, the time-dependent Schrödinger equation reads
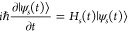
18This excitonic state can be expressed as a sum of time-independent states |α⟩
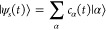
19with time-dependent coefficients *c*_α_(*t*). Additionally, the excitonic states |α⟩ can be written in terms of state |*m*⟩ localized at pigment *m* as
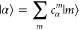
20

Furthermore, the probability density of finding an exciton on an individual pigment site m is given by
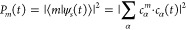
21

The dynamics of the probability density *P*_*m*_(*t*) reveals how an exciton moves and spreads over time among the different pigments. To obtain the probability density, the time-dependent Schrödinger equation, i.e., eq 18, is solved numerically iteratively using small time steps.^35,92^ The Hamiltonian is assumed to be constant during each time step necessitating the choice of a small enough time step. As a method for open quantum systems, the NISE approach requires statistical sampling. To this end, we averaged the calculations over 1000 realizations. However, it is computationally extremely expensive to calculate 1000 independent 6 ps-long trajectories used to determined the quantum dynamics for 6 ps. Instead, we used the site energies calculated by the TD-LC-DFTB along the 40 ps-long QM/MM MD trajectory of set 3 with a time step of 1 fs. This 40 ps-long trajectory was then split into the 1000 6 ps-long realizations by utilizing overlapping windows. While reducing the computational requirements drastically, the obvious drawback of this windowing approach is that the realizations are not completely independent but to a very good approximation. For the couplings, the averaged values from the 30,000 frames of the 3 μs-long MD trajectory were taken for all realizations.

**Figure 12:**
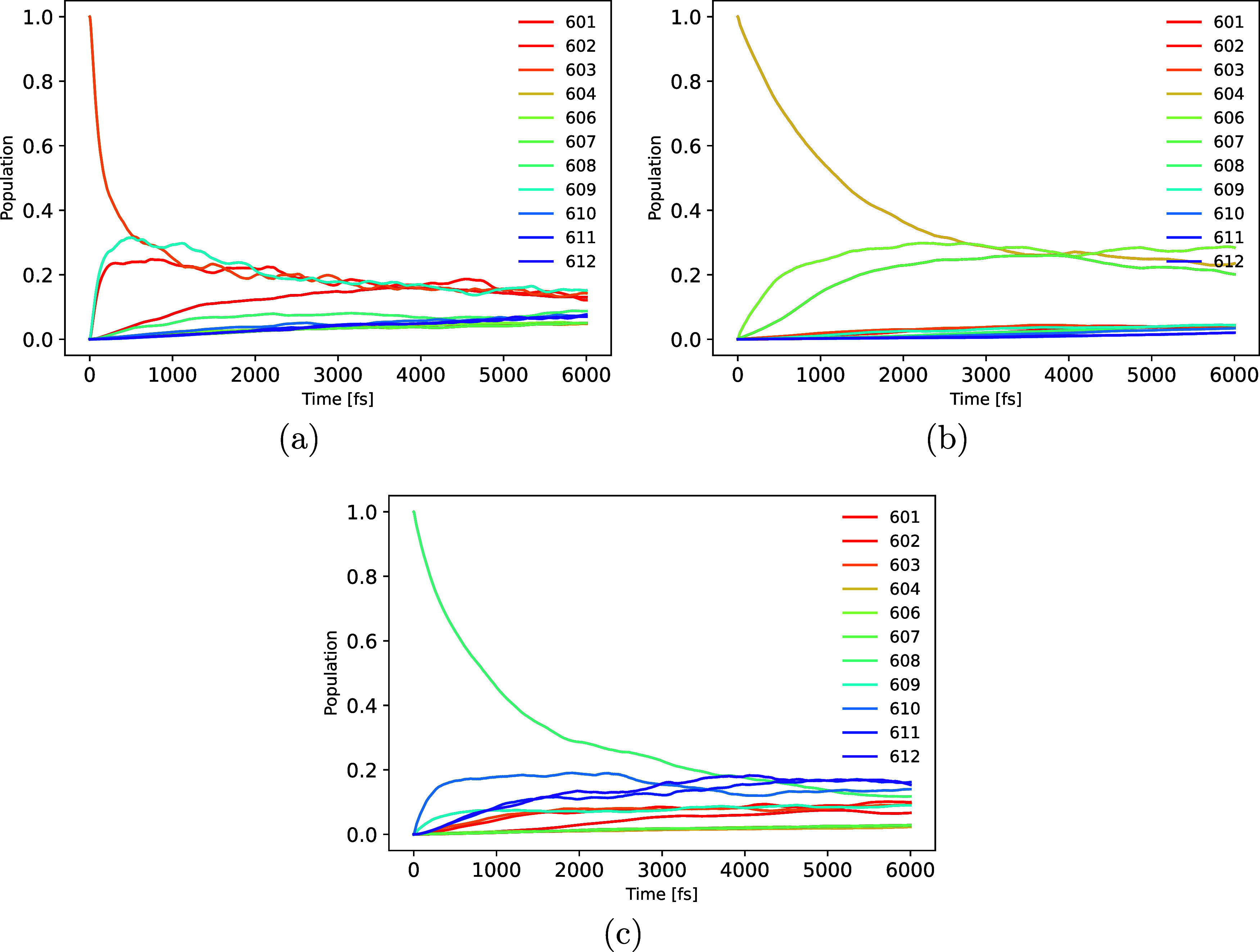
Exciton dynamics in the CP24 complex based on TD-LC-DFTB. While in panel (a) Chl-a 603 is initially excited, the initial excitations is on Chl-a 604 in panel (b) and on Chl-b 608 in panel (c).

To start the calculations, a single pigment is excited at a time and the propagation of excitation to the rest of the pigments in the CP24 complex is observed. Shown in Figure 12 is the population dynamics based on the initial excitation of pigments 603, 604 and 608, i.e., one from each of the three pigment clusters discussed above. The exciton dynamics starting at the rest of the pigments is depicted in Figure S12. On exciting the pigment Chl-a 601 in the cluster 601-602-603-609, no significant EET can be observed to pigments outside this cluster within 6000 fs. One has to note that the NISE approach does not lead to a proper thermal population at very long times but to an equal population of all sites. Excitation of the pigments 602, 603, and 609 leads to populating the other members of that particular cluster. Pigment Chl-a 603 exhibits the fastest decay, transferring the exciton to Chl-b 609 within the cluster. This is due to the highest coupling between these pigments. Exciting pigments 610, 611, and 612 from the cluster 608-610-611-612, the population more rapidly transfers to the remainder of the pigments. The excitation on Chl-b 608 decays slowly to Chl-a 612. The exciton relaxation dynamics for the individually excited pigments is happening on time scales between 1 and 5 ps, with several of them on the order of 3 ps. Notably, there was almost no transfer of excitons between the two clusters on the stromal side of the complex for over 6 ps. The luminal-side cluster is confirmed to be limited to exciton propagation among chlorophyll molecules 604, 606, and 607 with a slower exciton transfer compared to the two other clusters. As we observed earlier from the Hamiltonian and Mg–Mg distances, it is evident also from the exciton dynamics that three distinct clusters of chlorophyll molecules are present in the CP24 complex between which the exciton transfer is really slow. Even if such an intercluster transfer happens, it will be at time scales longer than 6 ps. Earlier, transient absorption experiments indicated that the exciton decay within the CP24 complex occurs within 3–5 ps when exciting the Chl-b molecules in the complex.^21^ In our calculations, exciton decay within the strongly coupled clusters of the CP24 complex also occurs within approximately 1–5 ps, with an average time of about 3 ps, reasonably well aligning with the experimental observations. An exciton relaxation dynamics on a similar time scale has also been reported for the CP29 complex.^93^

Already, based on the cryo-EM structure, energy transfer pathways were proposed based on the proximity of the chlorophyll molecules.^17^ The pigments Chl-b 608 and Chl-b 609 at the stromal side are expected to be involved in the EET to CP29. Also, on the luminal side, chlorophyll Chl-b 606 is rather close to Chl-b 614 of the CP29 complex. From the present results, it becomes clear that the most active energy transfer happens within the stromal side of the complex. Already previously, it was suggested that the energy transfer is presumably happening from LHCII and CP24 to CP29 based on the interpigment distances.^17^ Furthermore, ultrafast spectroscopic studies on the LHCII-CP29-CP24 complex indicate a rapid intermonomeric energy transfer between the CP24 and CP29 complexes.^23^ However, the dynamics observed in the present study refers to an isolated CP24 complex and is intramonomeric relaxation only. A more detailed picture of the EET pathways between the complexes requires the knowledge of the Hamiltonian of the merged complexes, e.g., LHCII-CP29-CP24.

## Conclusions

The minor antenna complex CP24 is a not too extensively studied light-harvesting complex in PSII, which is responsible for both EET and photoprotection in higher plants. In the present study, a multiscale QM/MM MD approach, which was previously used to analyze the light-harvesting properties of the complexes FMO, LHCII, CP29, and CP43,^34,35,44,47,82^ was extended to the CP24 complex. This investigation was based on a structure isolated from the cryo-EM structure of the *C*_2_*S*_2_*M*_2_-type PSII-LHCII supercomplex.^17^ For the first time, a 3 μs-long MD simulation was carried out to provide a comprehensive atomistic description of the CP24 complex within a POPC membrane. Based on a PCA, five conformations were extracted and used for generating DFTB/MM MD trajectories, effectively capturing the environmental fluctuations within the system. The five trajectories were subjected to the TD-LC-DFTB calculations to obtain the *Q*_*y*_ excitation energies of all 11 chlorophylls in the complex. The results were then analyzed in terms of average site energies including their fluctuations, site-dependent spectral densities and couplings. These quantities were further employed to generate the (time-dependent) Hamiltonian, which in turn was employed to determine the exciton dynamics and spectroscopic properties of the CP24 complex.

The application of DFTB/MM MD together with TD-LC-DFTB yields reliable site energies for the CP24 complex, aligning well with trends observed in previously reported site energies of similar complexes like LHCII and CP29.^27,94,95^ However, such comparisons have to be done with care since ultimately the spectroscopic properties of the unlike complexes are different due to variations in the proteins and slight differences in the pigment composition. The absorption spectrum of the CP24 complex computed using the FCE method is in reasonable agreement with the experimental counterpart. Again, this comparison has limitations since the employed cryo-EM structure and the complex used for the spectroscopic experiments originate from different organisms.^20^ Nevertheless, this comparison serves as a reasonable first step in validating our results against existing experimental observations. Furthermore, we determined the exciton transfer dynamics within the system substantiating the presence of three distinct chlorophyll clusters that likely facilitate the EET to neighboring complexes such as LHCII and CP29. The insights gained from the excitonic dynamics of the CP24 complex offer an opportunity to explore the EET mechanisms involving the outer LHCs of PSII in detail. On that note, it is to be highlighted that the Hamiltonian proposed here can potentially fill a gap in a recent computational study on ultrafast spectroscopy focusing on the larger complex LHCII-CP29-CP24.^23^ In that work, it was assumed that the CP24 site energies are equal to those in the LCHII complex which is certainly a reasonable first approximation but can be improved using the present results.

In addition to those results, the observed site energy shift across the various sets of DFTB/MM MD trajectories is of great interest. These shifts in site energies among identical chlorophyll molecules, observed across different starting structures are mainly due to movements in the local protein environment.^38,96^ The slow energy shifts induced by the environmental motions and conformational changes in protein contribute to what is known as static disorder.^43,97−99^ In addition to the changes in protein matrix, the present study revealed intriguing insights into the fluctuations in water molecule coordination to the Mg atoms in chlorophyll molecules. This observation was made possible through the long 3 μs MD simulation. While the protein environment primarily influences the changes in site energies through electrostatic effects, the role of changing the Mg coordination should not be overlooked in these discussions, as it can induce structural changes to the chlorophyll molecules, thereby influencing the site energies.^36,74,81,100−103^ As observed in the present study, these changes occur on time scales longer than the excitonic dynamics and are often not accounted for in short QM/MM MD trajectories or single-point calculations, though potentially introducing static disorder. Alternatively, one can model many copies of the same LHC in slightly different environments.^99^ All these findings underscore the necessity for conducting more extensive studies on LHCs and the impact of static disorder on their spectroscopic properties.

Overall, the characterization of the excitonic properties of the CP24 complex detailed in the current study represents another step toward achieving a better understanding of the energy funnel, starting from the outer LHCs to the reaction center in the PSII supercomplex. One intriguing perspective would be to explore the influence of intercomplex interactions on the site energies of CP24 within the multimeric light-harvesting complex LHCII-CP29-CP24, as well as to develop a comprehensive understanding of the full excitonic picture of the entire multimeric complex. As some of the challenges associated with studying larger systems gradually diminish thanks to the application of machine learning techniques,^104−107^ it is becoming easier to investigate larger LHCs and to compute their excitation properties in order to explore the corresponding EET. Recent advancements in multifidelity machine learning approaches show promise, as they become increasingly accurate in predicting the excitation energies of larger molecules.^107^ Enhancing our understanding of the energy transfer mechanisms in natural complexes using faster and more accurate tools lays the foundation for improving artificial LHCs.^3^

## Data Availability

The data that support the findings of this study are available from the corresponding author upon reasonable request.

## Supporting Information Available

RMSD plot of the protein chain, structural comparison of five frames, QM/MM representation of a chlorophyll molecule, the distribution of site energies, comparisons of CP24 site energies with the unshifted data of CP29 and LHCII, the individual spectral densities, individual contributions of pigments in each excited state, average Mg–Mg distances from the MD trajectory, the absorption and fluorescence spectra of the complex calculated using the Redfield approach with a clustering approach, the absorption spectra of the CP24 complex at 77 K by FCE, the exciton dynamics of additional pigments, a table listing the reorganization energies of all pigments of CP24, as well as a table representing the average dipole moment vectors (PDF)
