# Supplementary material for: Protein Effects on the Excitation Energies and Exciton Dynamics of the CP24 Antenna Complex
Source: J Phys Chem B. 2024 May 17;128(21):5201–17. doi: 10.1021/acs.jpcb.4c01637 (PMC11145653; doi:10.1021/acs.jpcb.4c01637)
Supplement: Supplementary file 1 — jp4c01637_si_001.pdf [file jp4c01637_si_001.pdf]

Supplementary Material: Protein Effects on the Excitation Energies and Exciton Dynamics of the CP24 Antenna Complex

Pooja Sarngadharan, Yannick Holtkamp, and Ulrich Kleinekathöfer*

School of Science, Constructor University, Campus Ring 1, 28759 Bremen, Germany

E-mail: ukleinekathoefer@constructor.university

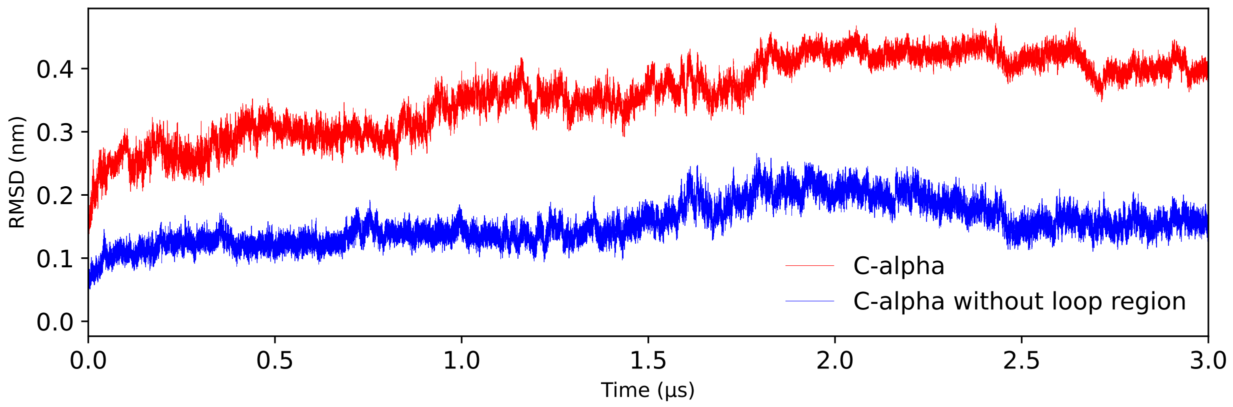

Figure S1: Comparison of root mean square deviation (RMSD) of the C-alpha atoms of the CP24 complex with and without the loop regions along the 3 μs-long classical MD trajectory.

Table S1: Reorganization energies of all 11 pigments of the CP24 complex, calculated from the individual spectral densities in units of eV.

**Pigments**	**b601**	**a602**	**a603**	**a604**	**b606**	**b607**	**b608**	**b609**	**a610**	**a611**	**a612**
λ_m	0.121	0.138	0.105	0.128	0.106	0.130	0.125	0.138	0.101	0.153	0.102

Table S2: Average dipole moment vector components (in Debye) used for the calculation of absorption and fluorescence spectra. The direction and magnitude of the transition dipole moments have been averaged separately. In addition, the magnitudes of the vectors are listed showing a clear variation of the average dipole moments among the different pigments.

**Pigments**	X	Y	Z	Magnitude
**b601**	-3.408	1.825	-3.579	5.27
**a602**	-3.868	-3.376	2.128	5.56
**a603**	5.724	0.805	-0.875	5.85
**a604**	1.870	-5.140	-2.265	5.92
**b606**	4.702	0.005	-1.943	5.09
**b607**	2.856	0.987	-4.294	5.25
**b608**	4.548	0.363	-0.934	4.66
**b609**	-3.731	-2.792	-0.388	4.68
**a610**	3.671	4.075	1.294	5.64
**a611**	4.690	2.814	-0.963	5.55
**a612**	-5.254	-1.684	1.594	5.74

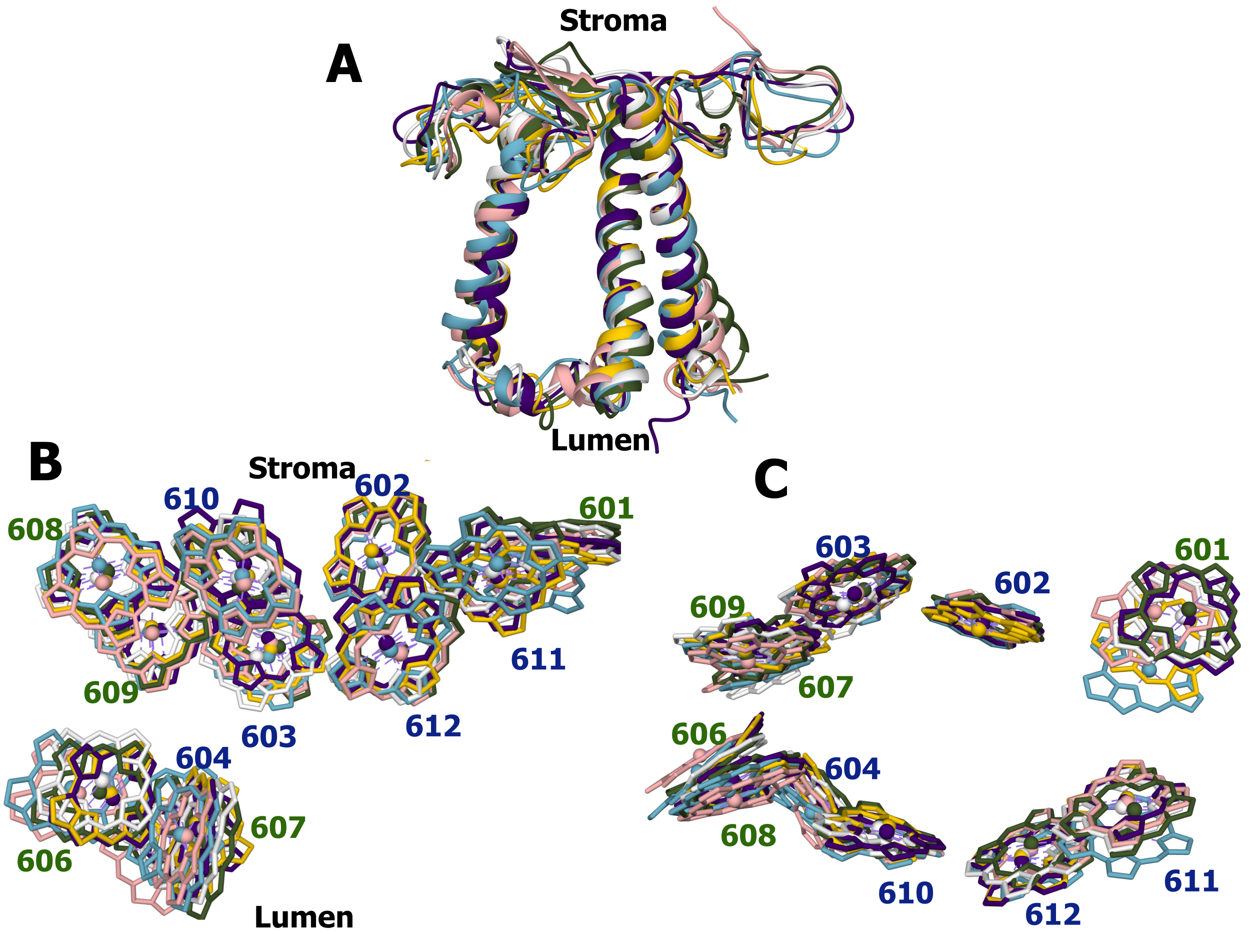

Figure S2: (A) Cartoon representations of protein chains illustrating the five initial conformations used for the QM/MM MD simulations overlaid to the cryo-EM structure. These conformations were determined based on a principal component analysis of the Mg-Mg distances derived from the 3 μs classical MD trajectory. The transmembrane helices exhibit minimal changes, while substantial fluctuations are observed in the loop regions. (B) Ball-and-stick models of the porphyrin rings in chlorophyll molecules, displayed in the same orientation as in (A), with all five structures superimposed on the cryo-EM structure. (C) Ball-and-stick models of the porphyrin rings in chlorophyll molecules, rotated by 90 degrees counterclockwise with respect to panel (B). In panels (B) and (C), the Chl-a and Chl-b molecules are labeled by their respective pigment numbers in blue and green, respectively. The conformational snapshots in A, B and C have been overlaid to the cryo-EM structure and are depicted in different colors: yellow (cryo-EM structure, PDB ID: 5XNL), violet (182.5 ns), light-blue (976.5 ns), pink (1754.5 ns), olive-green (2353.5 ns), and white (2887 ns) revealing the dynamic nature of the protein structure over time.

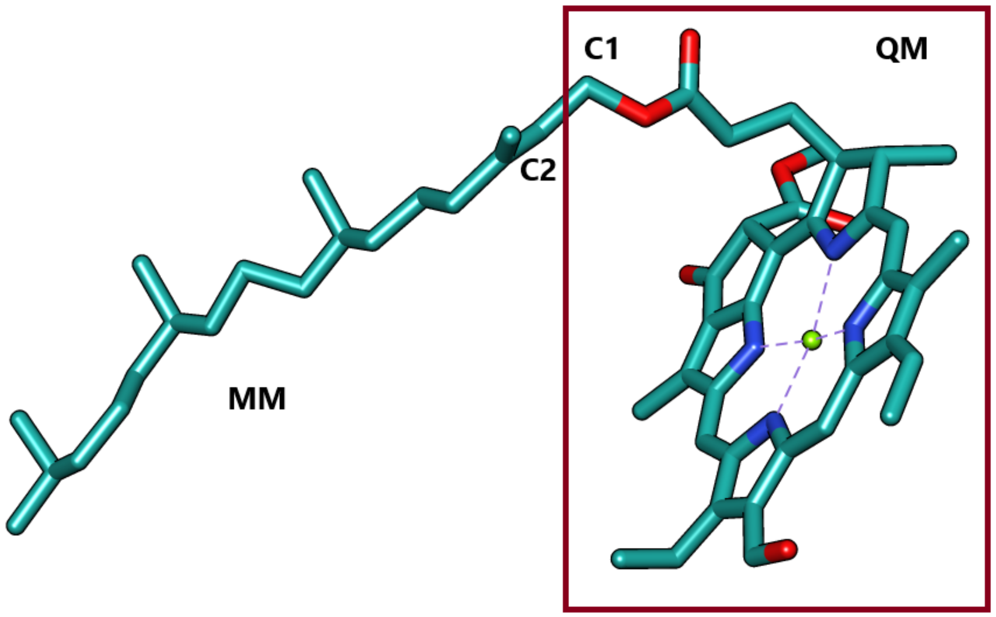

Figure S3: Stick representation of a Chl-a pigment showing the QM region and the MM regions. All atoms in the porphyrin ring up to the C1 atom in the phytyl chain are considered to be part of the QM region as indicated by the box. Atoms starting from atom C2 of the phytyl tail are part of the MM region. For Chl-a, the QM region comprises 82 atoms, whereas for Chl-b, it consists of 81 atoms. This difference arises because the methyl group in the porphyrin ring of Chl-a is replaced by an aldehyde group in Chl-b.

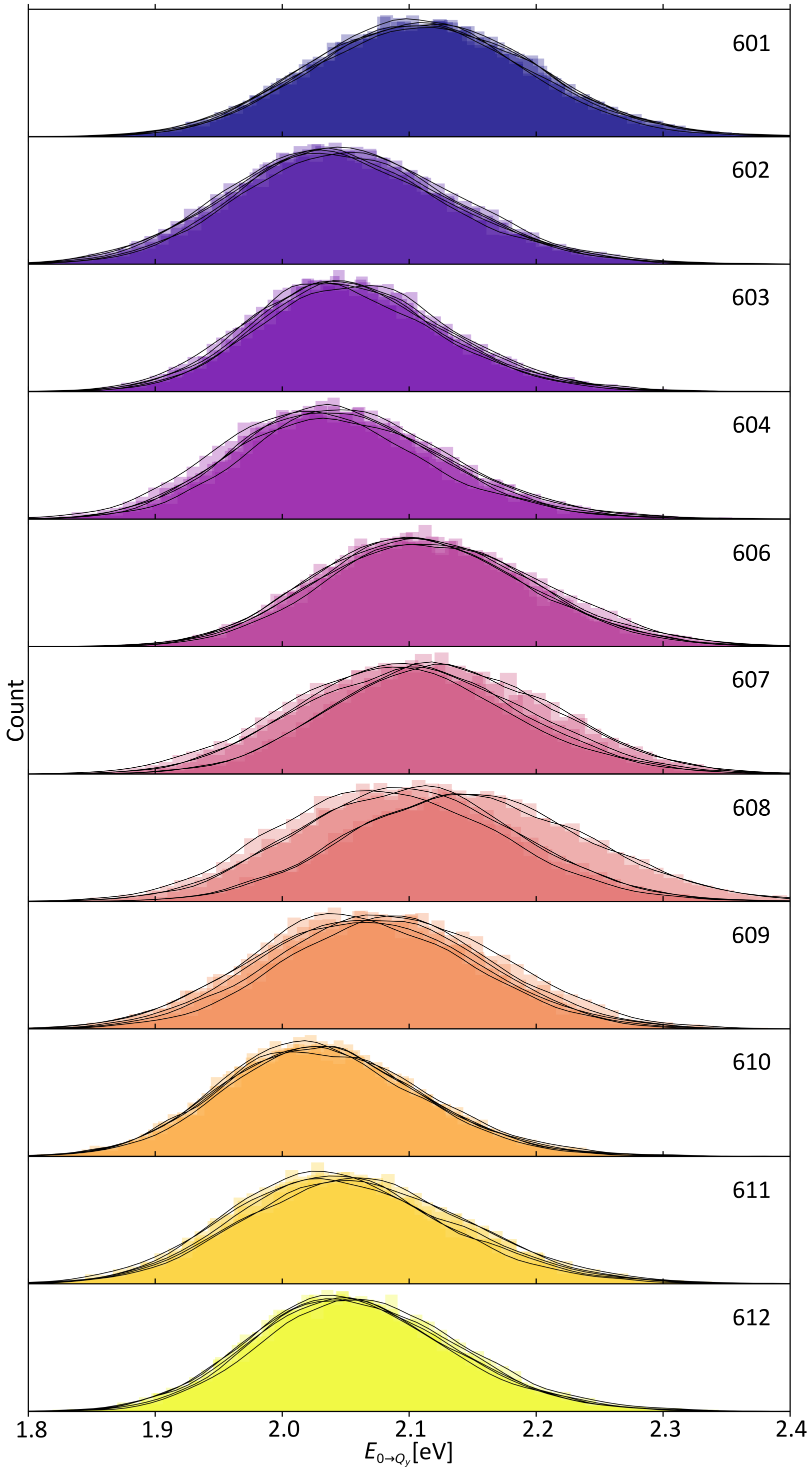

Figure S4: Distributions of the excitation energies for all chlorophyll molecules in the CP24 complex based on the TD-LC-DFTB approach along the five sets of 1 ns-long QM/MM MD trajectories. Each bin encompasses a range of equally divided excitation energies, and the height of each bin corresponds to the count of excitation energies falling into that particular range. The colored bins represent each pigment in the CP24 complex and all 5 sets of the same pigment are included. The black lines represent fits of the bins.

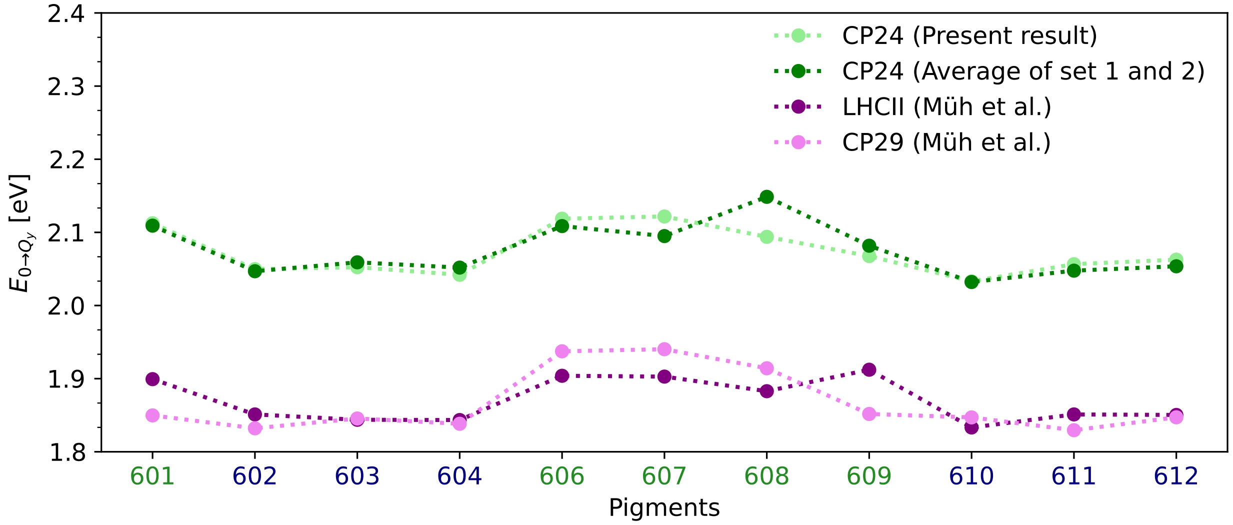

Figure S5: Average site energies of the pigments in the CP24 complex along the 1 ns-long trajectory using TD-LC-DFTB. The present findings (average of sets 3, 4 and 5) are compared to the site energies of the CP29 and LHCII complexes determined by Müh et al.[1] based on the crystal structure. The average of set 1 and 2 are also given for comparison.

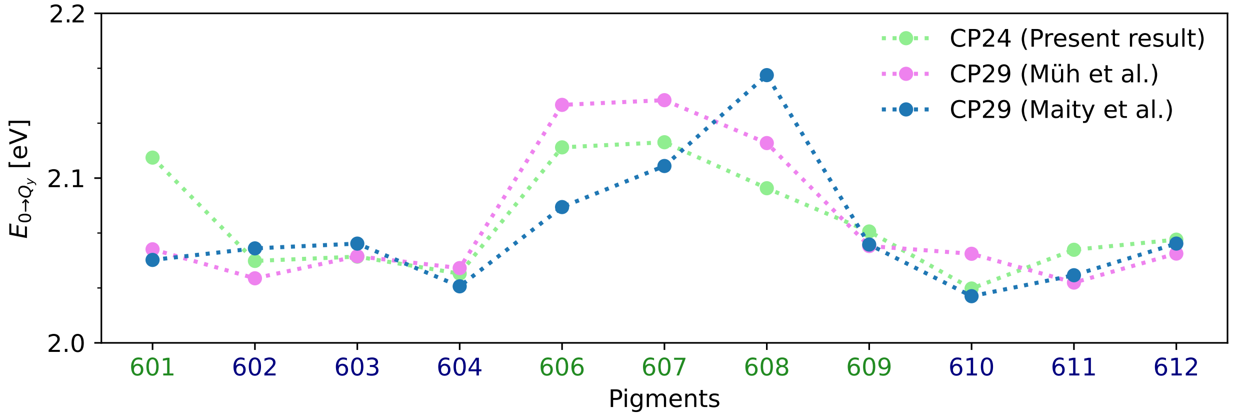

Figure S6: Average site energies of the pigments in the CP24 complex along the 1 ns-long trajectory using TD-LC-DFTB (average of sets 3, 4 and 5) are compared to the site energies of the CP29 complex (PDB ID: 3PL9) determined by the same method[2] but less sampling and the site energies of the CP29 determined by Müh et al.[1] based on the crystal structure. The CP29 energies by Müh et al. are shifted by 0.207 eV for better visual comparison.

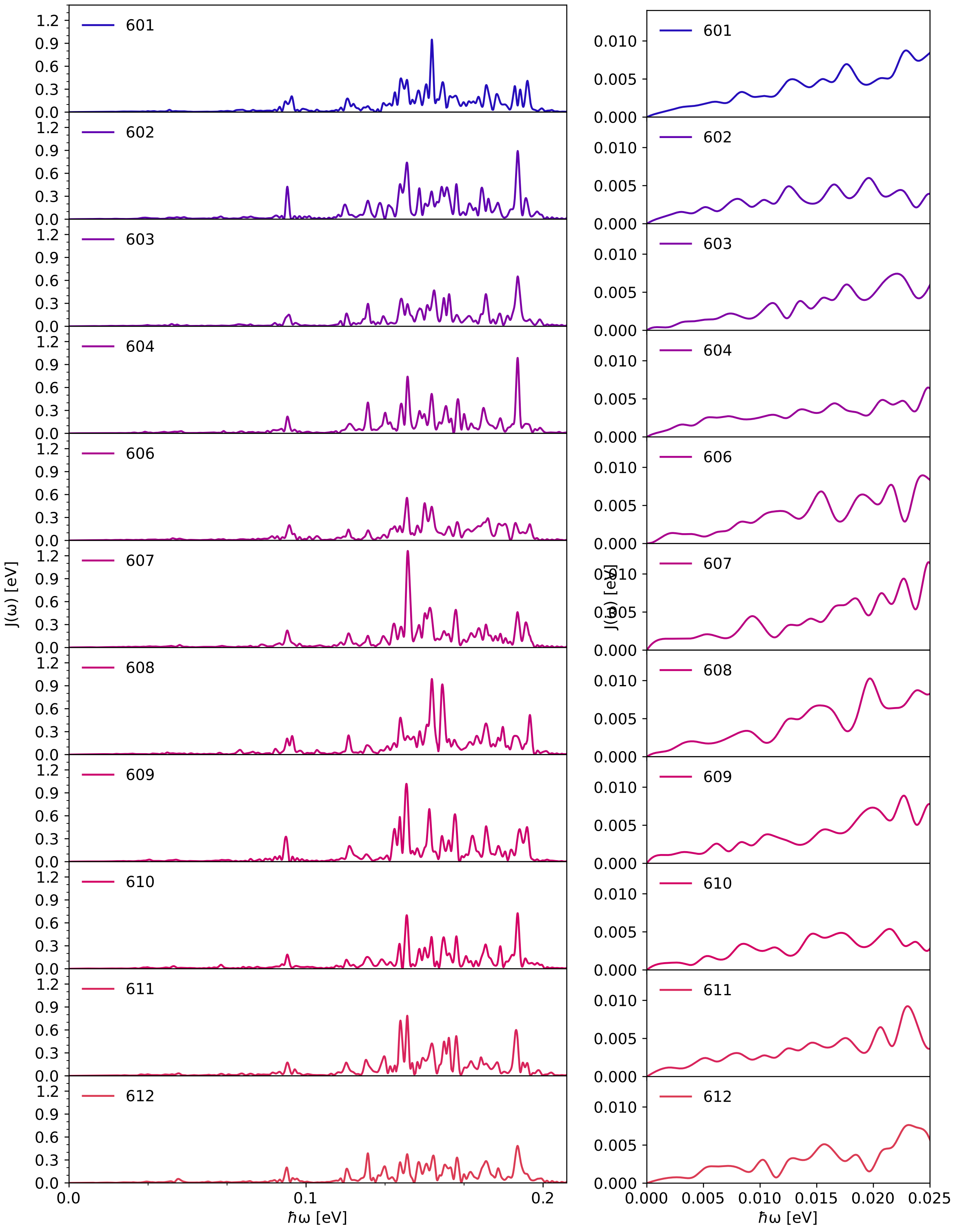

Figure S7: Calculated individual spectral densities of the 11 chlorophyll molecules in the CP24 complex, which were used as input for the calculations of the absorption and fluorescence spectra. On the right side, the low-frequency region is enlarged.

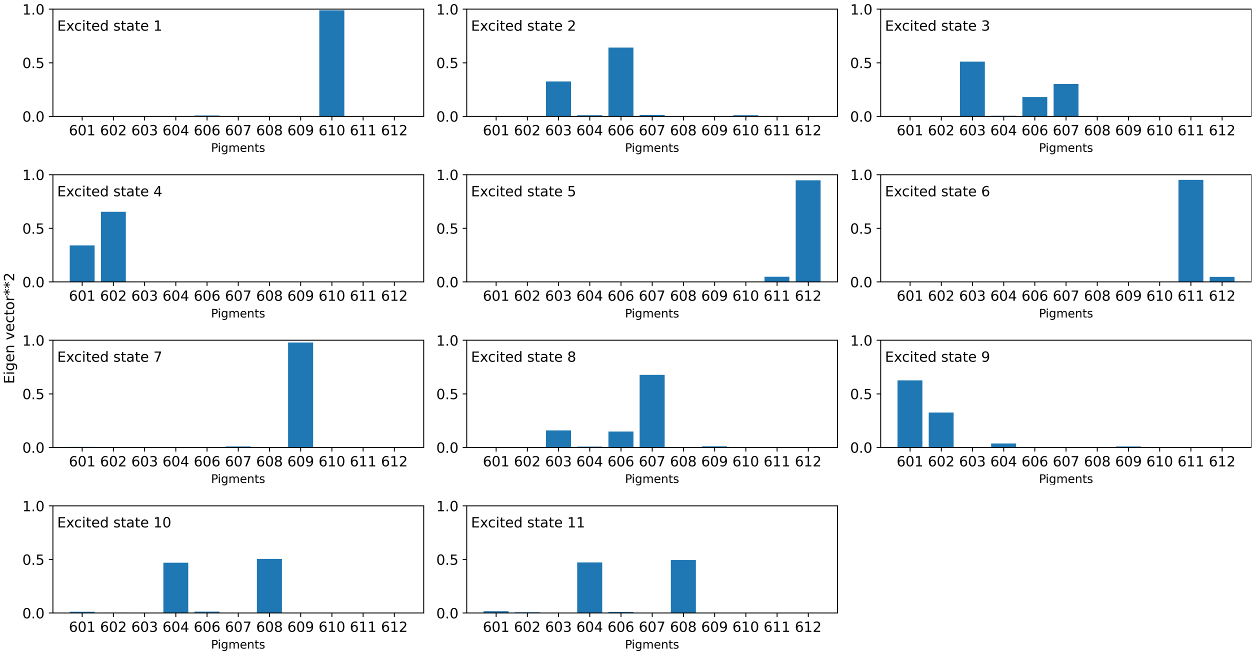

Figure S8: Individual contribution of the individual pigments to the excitonic states of the CP24 complex.

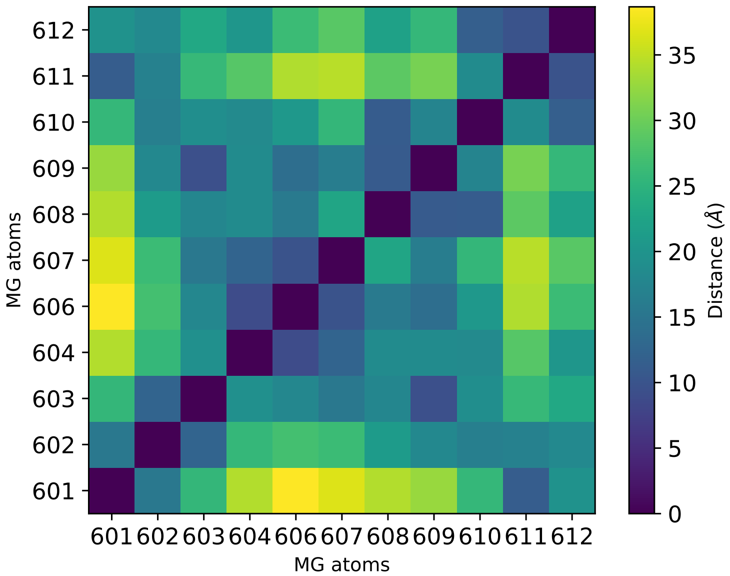

Figure S9: Average Mg-Mg distances between the chlorophyll molecules of the CP24 complex from the 3μs-long classical MD trajectory.

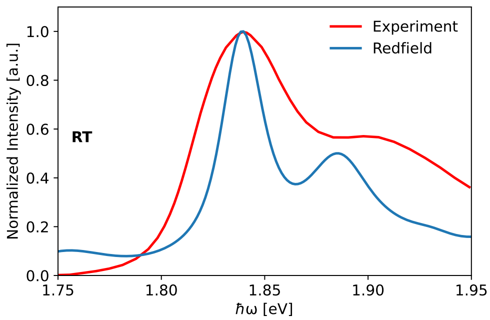

(a)

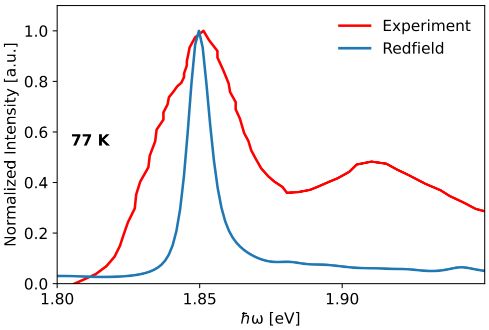

(b)

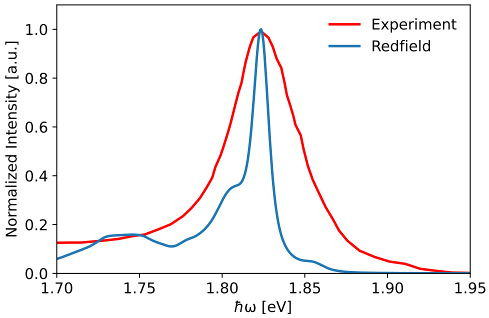

(c)

Figure S10: (a)Absorption spectra of complex CP24 at room temperature calculated using the Redfield approach but using three individual clusters (see main test). (b)Absorption spectra of complex CP24 at 77K calculated using three individual clusters. (c)Fluorescence spectra of complex CP24 at room temperature calculated using three individual clusters.

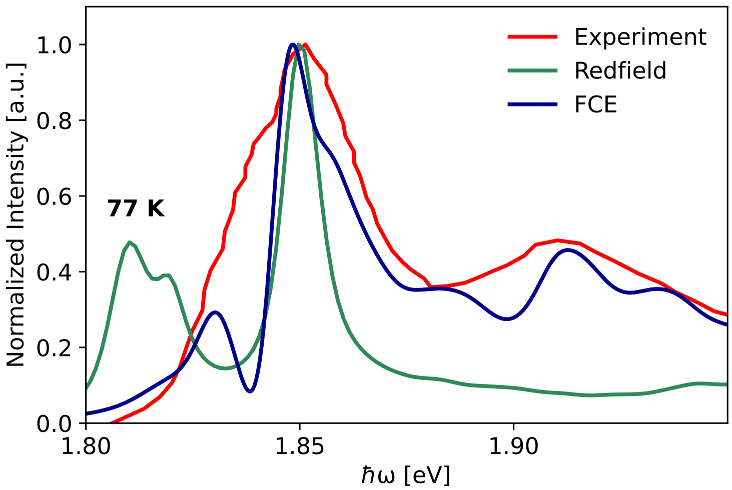

Figure S11: Absorption spectra of the CP24 complex based on the FCE and Redfield methods compared to the experimental spectrum at 77 K.[3] The FCE and the Redfield data have been shifted by 0.08 eV and 0.16 eV, respectively, toward lower energies to match the position of the main experimental peak.

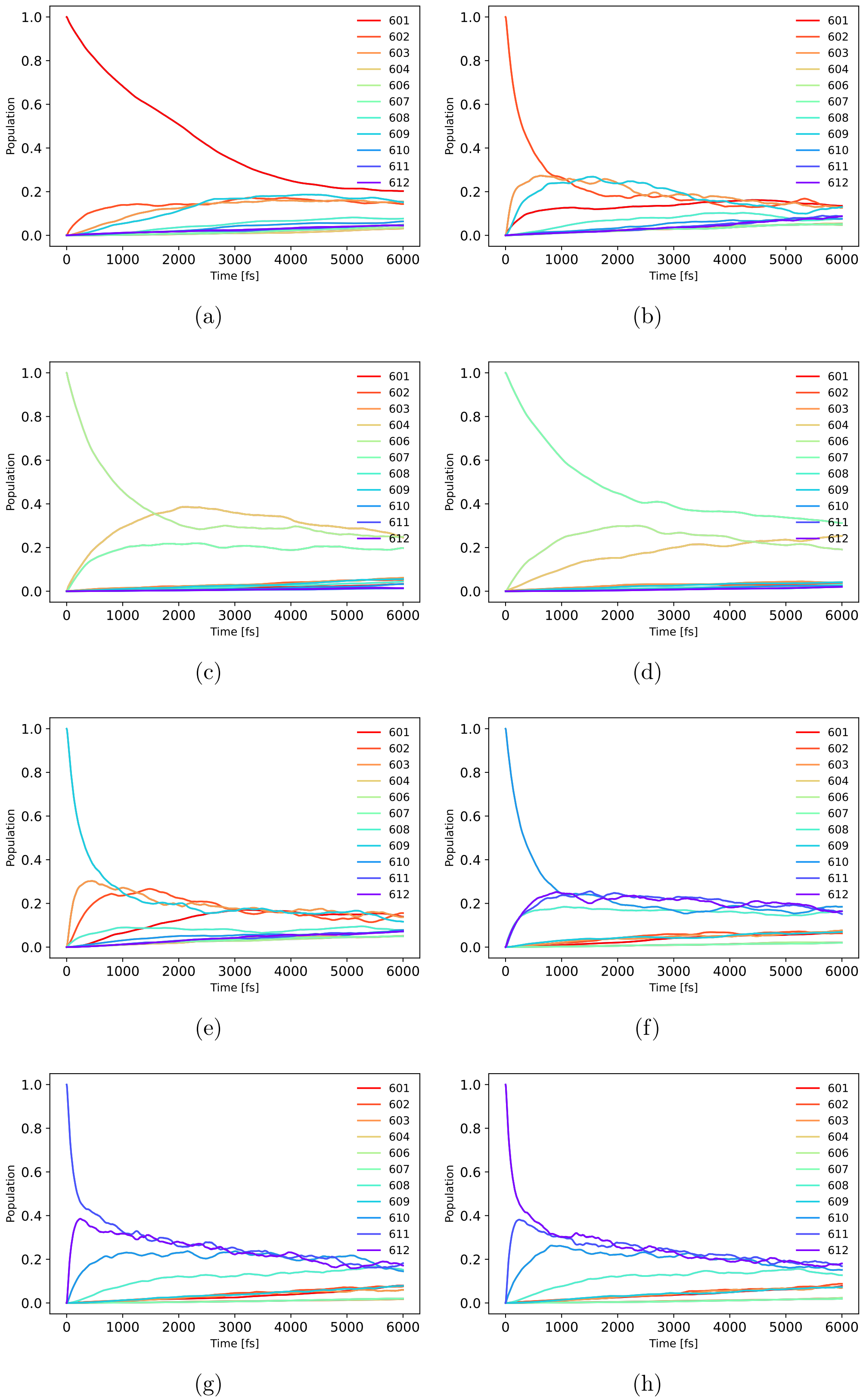

Figure S12: Exciton dynamics in the CP24 complex based on TD-LC-DFTB site energies from QM/MM MD trajectories and time-averaged couplings based on classical MD. Initially excited are (a) Chl-b 601, (b) Chl-a 602, (c) Chl-b 606, (d) Chl-b 607, (e) Chl-b 609, (f) Chl-a 610, (g) Chl-a 611, and (h) Chl-a 612.

References

(1) Müh, F.; Lindorfer, D.; am Busch, M. S.; Renger, T. Towards a Structure-Based Exciton Hamiltonian for the CP29 Antenna of Photosystem II. *Phys. Chem. Chem. Phys.* **2014**, *16*, 11848–11863, DOI: 10.1039/C3CP55166K.

(2) Maity, S.; Sarngadharan, P.; Daskalakis, V.; Kleinekathöfer, U. Time-Dependent Atomistic Simulations of the CP29 Light-Harvesting Complex. *J. Chem. Phys.* **2021**, *155*, 055103, DOI: 10.1063/5.0053259.

(3) Passarini, F.; Wientjes, E.; Hienerwadel, R.; Croce, R. Molecular Basis of Light Harvesting and Photoprotection in CP24. *J. Biol. Chem.* **2009**, *284*, 29536–29546, DOI: 10.1074/jbc.m109.036376.
